# Supplementary material for: Gene expression atlas for the food security crop cassava
Source: New Phytol. 2017 Jan 24;213(4):1632–41. doi: 10.1111/nph.14443 (PMC5516207; doi:10.1111/nph.14443)
Supplement: Supplementary file 1 — Fig. S1 Assessing variation among biological replicates. Fig. S2 Gene expression profile for Manes.09G108300. Fig. S3 Promoter:GUS fusion expression in Nicotiana benthamiana. Fig. S4 Identification of constitutively expressed genes and assessment of expression variation across sample type. [file NPH-213-1632-s001.pdf]

New Phytologist

***New Phytologist* Supporting Information**

Article title: **Gene expression atlas for the food security crop cassava.**

Authors: Mark C. Wilson, Andrew M. Mutka, Aaron W. Hummel, Jeffrey Berry, Raj Deepika Chauhan, Anupama Vijayaraghavan, Nigel J. Taylor, Daniel F. Voytas, Daniel H. Chitwood and Rebecca S. Bart

Article acceptance date: 18 December 2016

The following Supporting Information is available for this article:

Fig. S1 Assessing variation among biological replicates.

Fig. S2 Gene expression profile for Manes.09G108300.

Fig. S3 Promoter:GUS fusion expression in *Nicotiana benthamiana*.

Fig. S4 Identification of constitutively expressed genes and assessment of expression variation across sample type.

Notes S1 Documentation of data analysis (see separate file).

Notes S2 Promoter sequences used for GUS fusions (see separate file).

Notes S3 Genes differentially expressed between leaf and fibrous roots samples (see separate file).

Notes S4 Genes differentially expressed between OES and FEC samples (see separate file).

Notes S5 Genes upregulated in FEC tissue (see separate file).

Notes S6 Genes differentially expressed between storage roots and fibrous roots (see separate file).

Fig. S1 Assessing variation among biological replicates. (A) The squared coefficient of variation of replicates in each of the 11 sample types is shown here to be low and reasonably uniform across all sample types. This is indicative of replicates being closely related, limiting the possibility for error in sampling. **(B)** Distribution of all FPKM values greater than 1 in functionally annotated genes in each sample type plotted against a $\log_2$ scale on the *y*-axis. This demonstrates the similar expression of each sample type across annotated genes.

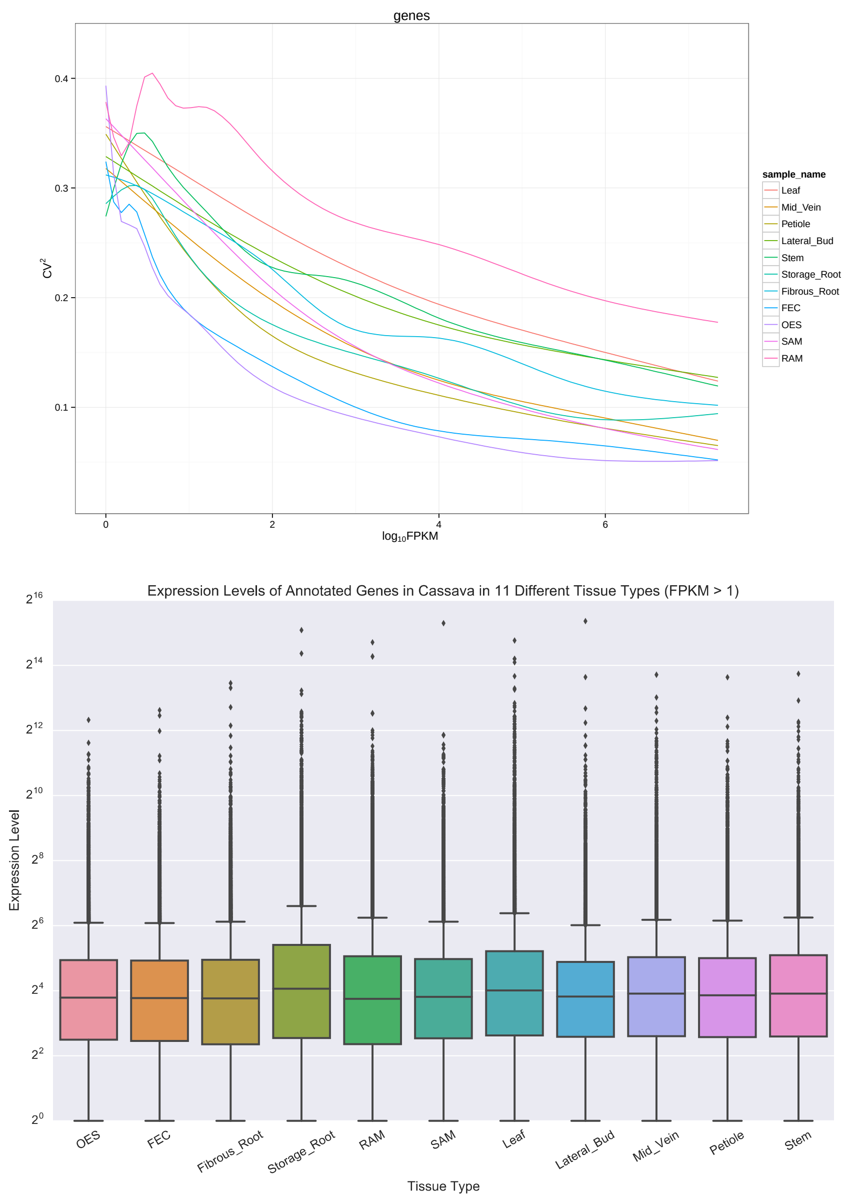

Fig. S2 Gene expression profile for Manes.09G108300. De Souza *et al*. have previously characterized the Pt2L4 gene (Manes.09G108300) and confirmed preferential expression in cassava storage roots but also in stems. This previously published expression pattern is consistent with the current dataset, as displayed.

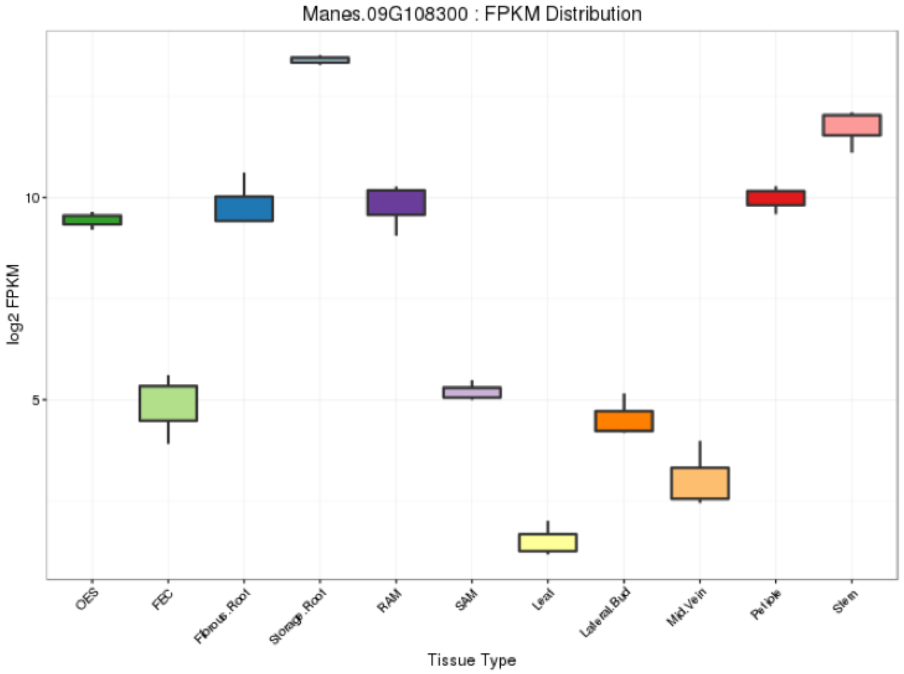

Fig. S3 Promoter:GUS fusion expression in *Nicotiana benthamiana*. Ability of promoters to drive gene expression was assessed transiently in *Nicotiana benthamiana*. *Agrobacterium* was used to delivery binary vectors containing each promoter fused to the GUS reporter gene.

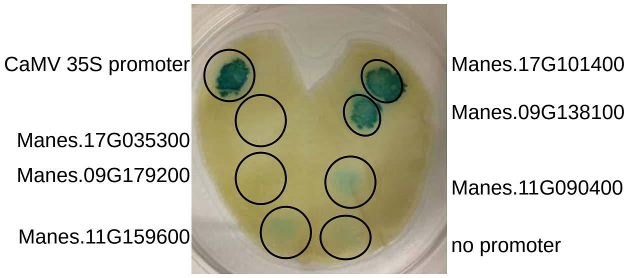

Fig. S4 Identification of constitutively expressed genes and assessment of expression variation across sample type. Expression profile across 11 sample types was investigated for three housekeeping genes: GTPb, PP2A and UBQ10. The dataset was queried for genes that showed medium level expression (> 40 FPKM) and low variability (low coefficient of variation) across all samples. Top 10 genes are displayed.

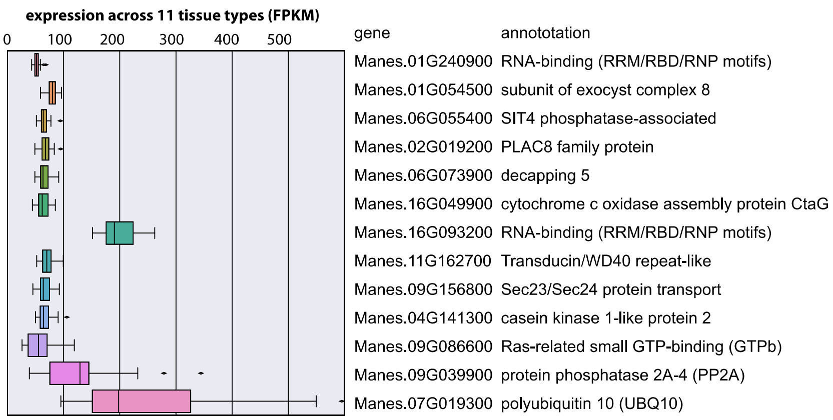

Notes S1 Documentation of data analysis. Custom Python code was used in a Jupyter notebook using the Pandas, NumPy, Seaborn, and SciPy packages to organize, process, and display the data (see separate file).

Notes S2 Promoter sequences used for GUS fusions. Promoter fragments, listed in Note S2, were cloned from cassava variety TME419 into a pCAMBIA vector upstream of GUS (see separate file).

Notes S3 Genes differentially expressed between leaf and fibrous roots samples. List contains genes differentially expressed greater than 4-fold ($|\log_2(\text{fold\_change})| > 2$) (see separate file).

Notes S4 Genes differentially expressed between OES and FEC samples. Over 2000 genes were identified as differentially expressed between OES and FEC samples greater than 4-fold ($|\log_2(\text{fold\_change})| > 2$) (see separate file).

Notes S5 Genes upregulated in FEC tissue. Annotations suggest that genes are associated with sulfur and sulfate transport (see separate file).

Notes S6 Genes differentially expressed between storage roots and fibrous roots. List contains genes differentially expressed > 4-fold ($|\log_2(\text{fold\_change})| > 2$) (see separate file).
