# Supplementary material for: Gene expression atlas for the food security crop cassava
Source: New Phytol. 2017 Jan 24;213(4):1632–41. doi: 10.1111/nph.14443 (PMC5516207; doi:10.1111/nph.14443)
Supplement: Supplementary file 2 — Notes S1 Documentation of data analysis. [file NPH-213-1632-s002.pdf]

# tissue_specific_rnaseq_analysis_jupyter_notebook

December 19, 2016

## 1 Supporting Information Notes S1. Documentation of data analysis.

Custom Python code was used in a Jupyter notebook using the Pandas, NumPy, Seaborn, and SciPy packages to organize, process, and display the data.

#### 1.0.1 Files Required in this Notebook and their Source:

| Purpose | File | Source |
|---|---|---|
| Annotation | Mesculenta_305_v6.1.annotation_info.txt | Phytozome10.3 |
| Gene Expression | gene_exp.diff | Cuffdiff |
| Gene Expression | genes.read_group_tracking | Cuffdiff |
| GO Enrichment | 4.genes_nodes_mean_exp.txt | SOM Clustering Analysis |

```
In [1]: import sys,os

        import pandas as pd                  # version 0.17.0
        import numpy as np                   # version 1.11.0
        import seaborn as sns                # version 0.7.0
        import matplotlib.pyplot as plt      # version 1.5.1

        from scipy.stats import percentileofscore as percentileofscore
        # version 0.16.0

        %matplotlib inline
```

```
In [2]: os.chdir('tissue_specific_rnaseq/')
```

### 1.1 Annotation

```
In [3]: # read in annotations for AM560-2 assembly version 6.1
        annot = pd.read_table( 'Mesculenta_305_v6.1.annotation_info.txt',
                               sep='\t', header=None )

        # limit table to specific columns
        #    and drop any duplicates that may be present
        annot = annot[[1,5,9,10,12]].drop_duplicates(subset=1).rename(
            columns={1:'gene',5:'PTHR',9:'go',10:'TAIR',12:'annot'})

        # for the genes that do not have an arabidopsis functional annotation,
        #     use the PANTHER funtional annotation provided where possible
        annot['annot'][annot['annot'].isnull()] = annot['PTHR']
```

```
# drop the PANTHER column
annot = annot.dropna(axis=0, subset=['annot']).drop(['PTHR'], axis=1)

df_go = annot[['go','gene']].dropna().set_index('gene')['go']
df_go = df_go.str.split(',').apply(pd.Series,1).stack()
df_go.index = df_go.index.droplevel(-1)
df_go.name = 'go'

annot.columns
```

Out[3]: Index(['gene', 'go', 'TAIR', 'annot'], dtype='object')

In [4]:
```
print('Annotated Gene Count: {}'.format(annot.shape[0]) )
```

Annotated Gene Count: 26015

## 1.2 Read in Data

In [5]:
```
# read in data
df_cuff = pd.read_table( 'gene_exp.diff',
                        sep='\t', index_col=0)

# Create dataframe to hold all duplicated genes (defined as
#    genes containing)
df_dups = df_cuff.drop_duplicates(['gene_id','gene'])['gene']
df_shiny = df_dups.str.split(',').apply(pd.Series,1).stack()

df_dups = df_dups[df_dups.str.contains(',')
                 ].str.split(',').apply(pd.Series,1).stack()
df_dups.index = df_dups.index.droplevel(-1)
df_shiny.index = df_shiny.index.droplevel(-1)
df_shiny.name = 'gene'


df_cuff['gene'] = df_cuff['gene'].str.replace(r',.*', '')

df_cuff.columns
```

Out[5]:
```
Index(['gene_id', 'gene', 'locus', 'sample_1', 'sample_2', 'status', 'value_1',
       'value_2', 'log2(fold_change)', 'test_stat', 'p_value', 'q_value',
       'significant'],
      dtype='object')
```

In [6]:
```
# create dataframe containing each gene in one row with its
#    expression values in each tissue type
df_cuff_exp = pd.concat([
        df_cuff[['gene_id',
                 'gene',
                 'locus',
                 'sample_1',
                 'value_1']].rename(columns={'sample_1':'sample',
                                            'value_1':'value'}),
```

```
            df_cuff[['gene_id',
                     'gene',
                     'locus',
                     'sample_2',
                     'value_2']].rename(columns={'sample_2':'sample',
                                                 'value_2':'value'}),
        ]).drop_duplicates().pivot(index='gene_id',
                                   columns='sample',
                                   values='value')

    # reorganize column names with similar tissues near each other
    tissue_order = ['Leaf','Mid_Vein','Petiole','Stem','Lateral_Bud','SAM',
                    'Storage_Root','Fibrous_Root','RAM',
                    'OES','FEC'
                   ]
    df_cuff_exp = df_cuff_exp.loc[:,tissue_order]

    df_cuff_exp.columns
```

Out[6]:
```
Index(['Leaf', 'Mid_Vein', 'Petiole', 'Stem', 'Lateral_Bud', 'SAM',
       'Storage_Root', 'Fibrous_Root', 'RAM', 'OES', 'FEC'],
      dtype='object', name='sample')
```

In [7]:
```
# remove all rows with all 0's, with no annotation
# merge expression data with annotation data and
#    only keep genes that have been annotated
df_cuff_ann_all = df_cuff_exp.merge(
    df_cuff[['gene_id','gene','locus']],
    left_index=True,
    right_on='gene_id',
    copy=False).drop_duplicates()

# drop all denovo genes
df_cuff_ann_all = df_cuff_ann_all[df_cuff_ann_all['gene'] != '-']

# add annotation (gene names)
df_cuff_ann_all = df_cuff_ann_all.merge( annot[['gene','annot']],
                                        how='left',
                                        on='gene',
                                        copy=False)

# drop all genes that do not have an annotation
df_cuff_ann = df_cuff_ann_all.dropna()

# drop genes with lower than 0 mean expression
df_cuff_ann = df_cuff_ann[df_cuff_ann.mean(axis=1) > 0]
df_cuff_ann.columns
```

Out[7]:
```
Index(['Leaf', 'Mid_Vein', 'Petiole', 'Stem', 'Lateral_Bud', 'SAM',
       'Storage_Root', 'Fibrous_Root', 'RAM', 'OES', 'FEC', 'gene_id', 'gene',
       'locus', 'annot'],
      dtype='object', name='sample')
```

In [8]:
```
# read data from genes.read_group_tracking file
genes_rgt = pd.read_table('genes.read_group_tracking')
```

```
# merge condition and replicate columns to pivot table on
genes_rgt['pivot'] = genes_rgt['condition'] + genes_rgt['replicate'].astype(str)

# pivot table to look at expression values for each replicate per gene
genes_rgt_piv = genes_rgt.pivot(index='tracking_id',
                                columns='pivot',
                                values='FPKM')


# merge with gene names
genes_rgt_piv = genes_rgt_piv.merge(df_cuff.loc[:,['gene_id',
                                                    'gene'
                                                   ]].drop_duplicates(),
                                    left_index=True,
                                    right_on='gene_id')

# Merge with functional annotations
genes_rgt_piv = genes_rgt_piv.merge( annot,
                                    on='gene',
                                    how='left' ).set_index('gene_id')

genes_rgt_piv['gene_id'] = genes_rgt_piv.index

# for later analyses, prep tissue to index dictionary
tissue_rep_index = {'FEC':[0,1,2],
                    'Fibrous_Root':[3,4,5],
                    'Lateral_Bud':[6,7,8],
                    'Leaf':[9,10,11],
                    'Mid_Vein':[12,13,14],
                    'OES':[15,16,17],
                    'Petiole':[18,19,20],
                    'RAM':[21,22,23],
                    'SAM':[24,25,26],
                    'Stem':[27,28,29],
                    'Storage_Root':[30,31]
                   }

genes_rgt_piv.columns
```

Out[8]:
```
Index(['FEC0', 'FEC1', 'FEC2', 'Fibrous_Root0', 'Fibrous_Root1',
       'Fibrous_Root2', 'Lateral_Bud0', 'Lateral_Bud1', 'Lateral_Bud2',
       'Leaf0', 'Leaf1', 'Leaf2', 'Mid_Vein0', 'Mid_Vein1', 'Mid_Vein2',
       'OES0', 'OES1', 'OES2', 'Petiole0', 'Petiole1', 'Petiole2', 'RAM0',
       'RAM1', 'RAM2', 'SAM0', 'SAM1', 'SAM2', 'Stem0', 'Stem1', 'Stem2',
       'Storage_Root0', 'Storage_Root1', 'gene', 'go', 'TAIR', 'annot',
       'gene_id'],
      dtype='object', name='pivot')
```

In [9]:
```
#########################
# RSHINY APP FILE PREP ##
# print files for RShiny App
df_temp = genes_rgt_piv[genes_rgt_piv.gene != '-'].drop_duplicates().copy()
del df_temp['gene']
df_temp = df_temp.merge( pd.DataFrame(df_shiny),
```

```
                                      how='left',
                                      left_index=True,
                                      right_index=True )

        df_temp['possible_issues'] = df_temp['gene'].isin(df_dups)

        print(df_temp.shape)
        df_temp.to_csv('mesculenta_v6_output/Rshiny_app_dataset.txt',
                       sep='\t', index=False)


        ## Print genes with multiple associated annotations
        df_dups.to_csv('mesculenta_v6_output/warning_genes.txt',
                       index=False, header=False)
        print( df_dups.shape )
```

```
(35200, 38)
(4531,)
```

# 2 SOM Clustering with GO Enrichment

## 2.1 Find all genes with at least one significantly differentially expressed pairwise comparison

This data is for use in an analysis in R to identify gene expression clusters using a self organizing map

```
In [10]: # write to file all gene names with a statistically significant pairwise
         #    difference where one of the pairs is expressed greater than 1 FPKM
         df_gene_ids = pd.DataFrame(df_cuff[(df_cuff['q_value'] <= 0.05)
                                  & ((df_cuff['value_1'] > 1) | (df_cuff['value_2'] > 1))
                                 ].loc[:,['gene_id','gene']].drop_duplicates().index)
         df_gene_ids.to_csv('mesculenta_v6_output/0.cassava_diff_genes.txt',
                            index=False, sep='\t')
```

```
In [11]: # write to file all genes with at least one tissue expressed
         #     greater than 1 FPKM for use as background in the GO analysis
         # print to file expression values for all genes expressed > 1FPKM
         genes_rgt_piv.iloc[:,:32].to_csv('./mesculenta_v6_output/1.cassava_exp.txt',
                                          sep='\t')

         # print to file expression values with functional annotations
         genes_rgt_piv.drop('go', axis=1
                           ).to_csv('./mesculenta_v6_output/2.cassava_annotation.txt',
                                          sep='\t')
```

# 3 Parse and Trim goatools GO Terms from R Cluster Analysis

A python tool called goatools was used to create the files being read in this section. The 4 nodes are based on a self organizing map from an R analysis to cluster genes by expression profile

#### 3.0.1 GO PREP

**Split Genes into Files by node**

```
In [12]: df_pcanodes = pd.read_table('4.genes_nodes_mean_exp.txt',
                                     sep='\t', usecols = ['test_id','node'])

         print(df_pcanodes.shape)
```

```
(14426, 2)
```

```
In [13]: ## READ IN NODE DATA FROM DAN'S ANALYSIS
         df_node1 = df_pcanodes[df_pcanodes['node'] == 1
                               ].merge(df_cuff.loc[:,['gene']].drop_duplicates(),
                                       left_on='test_id',
                                       right_index=True
                                      )
         df_node2 = df_pcanodes[df_pcanodes['node'] == 2
                               ].merge(df_cuff.loc[:,['gene']].drop_duplicates(),
                                       left_on='test_id',
                                       right_index=True
                                      )
         df_node3 = df_pcanodes[df_pcanodes['node'] == 3
                               ].merge(df_cuff.loc[:,['gene']].drop_duplicates(),
                                       left_on='test_id',
                                       right_index=True
                                      )
         df_node4 = df_pcanodes[df_pcanodes['node'] == 4
                               ].merge(df_cuff.loc[:,['gene']].drop_duplicates(),
                                       left_on='test_id',
                                       right_index=True
                                      )

         ## PRINT NODE COUNTS TO SCREEN
         print('node1 counts: {}'.format(df_node1.shape[0]) )
         print('node2 counts: {}'.format(df_node2.shape[0]) )
         print('node3 counts: {}'.format(df_node3.shape[0]) )
         print('node4 counts: {}'.format(df_node4.shape[0]) )

         ## PRINT GO PREP GENE NAMES TO FILE
         df_cuff[(df_cuff['q_value'] <= 0.05)
                 & ((df_cuff['value_1'] > 1) | (df_cuff['value_2'] > 1))
                ].loc[:,['gene_id','gene']
                     ].drop_duplicates()['gene'].to_csv('goprep_pcasom_bkgrnd.txt',
                                                         index=False, sep='\t' )

         df_cuff[((df_cuff['value_1'] > 1) | (df_cuff['value_2'] > 1))
                ].loc[:,['gene_id','gene']
                     ].drop_duplicates()['gene'].to_csv('goprep_pcasom_bkgrnd_all.txt',
                                                         index=False, sep='\t' )


         df_node1['gene'].to_csv('goprep_pcasom_node1.txt',
                                 index=False, sep='\t' )
         df_node2['gene'].to_csv('goprep_pcasom_node2.txt',
                                 index=False, sep='\t' )
         df_node3['gene'].to_csv('goprep_pcasom_node3.txt',
```

```
                                index=False, sep='\t' )
        df_node4['gene'].to_csv('goprep_pcasom_node4.txt',
                                index=False, sep='\t' )
```

```
node1 counts: 2672
node2 counts: 2672
node3 counts: 2914
node4 counts: 3727
```

### 3.0.2 NODE1

```
In [14]: node1_df = pd.read_table('pcasom_node1_goatools_noprop.txt', skiprows=2)

         # GO Term list before limiting by significance
         print('Before significance filtering')
         print('GO Count: {}'.format(node1_df.shape[0]))
         print('Fraction of enriched genes: {:.2f}'.format(
                 node1_df[node1_df['enrichment'] == 'e'].shape[0]/node1_df.shape[0]))

         # limit GO Terms by FDR corrected p value
         node1_df = node1_df[node1_df['p_fdr'] < 0.001]

         # GO Term list before limiting by significance
         print('\nAfter significance filtering')
         print('GO Count: {}'.format(node1_df.shape[0]))
         print('Fraction of enriched genes: {:.2f}'.format(
                 node1_df[node1_df['enrichment'] == 'e'].shape[0]/node1_df.shape[0]))

         node1_df = node1_df[node1_df['enrichment'] == 'e']

         node1_df.to_csv('pcasom_node1_goenrichment.txt', sep='\t', index=False)

         df_node1_gotags = node1_df.loc[:,'id']

         node1_df.columns
```

```
Before significance filtering
GO Count: 155
Fraction of enriched genes: 0.84

After significance filtering
GO Count: 35
Fraction of enriched genes: 0.80
```

```
Out[14]: Index(['id', 'enrichment', 'description', 'ratio_in_study', 'ratio_in_pop',
                'p_uncorrected', 'p_bonferroni', 'p_holm', 'p_sidak', 'p_fdr'],
               dtype='object')
```

### 3.0.3 NODE2

```
In [15]: node2_df = pd.read_table('pcasom_node2_goatools_noprop.txt', skiprows=2)
```

```
# GO Term list before limiting by significance
print('Before significance filtering')
print('GO Count: {}'.format(node2_df.shape[0]))
print('Fraction of enriched genes: {:.2f}'.format(
        node2_df[node2_df['enrichment'] == 'e'].shape[0]/node2_df.shape[0]))

# limit GO Terms by FDR corrected p value
node2_df = node2_df[node2_df['p_fdr'] < 0.01]

# GO Term list before limiting by significance
print('\nAfter significance filtering')
print('GO Count: {}'.format(node2_df.shape[0]))
print('Fraction of enriched genes: {:.2f}'.format(
        node2_df[node2_df['enrichment'] == 'e'].shape[0]/node2_df.shape[0]))

node2_df = node2_df[node2_df['enrichment'] == 'e']

node2_df.to_csv('pcasom_node2_goenrichment.txt', sep='\t', index=False)

df_node2_gotags = node2_df.loc[:,'id']

node2_df.columns
```

```
Before significance filtering
GO Count: 84
Fraction of enriched genes: 0.58

After significance filtering
GO Count: 6
Fraction of enriched genes: 0.50
```

```
Out[15]: Index(['id', 'enrichment', 'description', 'ratio_in_study', 'ratio_in_pop',
               'p_uncorrected', 'p_bonferroni', 'p_holm', 'p_sidak', 'p_fdr'],
              dtype='object')
```

### 3.0.4 NODE3

```
In [16]: node3_df = pd.read_table('pcasom_node3_goatools_noprop.txt', skiprows=2)

         # GO Term list before limiting by significance
         print('Before significance filtering')
         print('GO Count: {}'.format(node3_df.shape[0]))
         print('Fraction of enriched genes: {:.2f}'.format(
                 node3_df[node3_df['enrichment'] == 'e'].shape[0]/node3_df.shape[0]))

         # limit GO Terms by FDR corrected p value
         node3_df = node3_df[node3_df['p_fdr'] < 0.001]

         # GO Term list before limiting by significance
         print('\nAfter significance filtering')
         print('GO Count: {}'.format(node3_df.shape[0]))
         print('Fraction of enriched genes: {:.2f}'.format(
                 node3_df[node3_df['enrichment'] == 'e'].shape[0]/node3_df.shape[0]))
```

```
node3_df = node3_df[node3_df['enrichment'] == 'e']

node3_df.to_csv('pcasom_node3_goenrichment.txt', sep='\t', index=False)

df_node3_gotags = node3_df.loc[:,'id']

node3_df.columns
```

```
Before significance filtering
GO Count: 92
Fraction of enriched genes: 0.71

After significance filtering
GO Count: 10
Fraction of enriched genes: 0.60
```

```
Out[16]: Index(['id', 'enrichment', 'description', 'ratio_in_study', 'ratio_in_pop',
                'p_uncorrected', 'p_bonferroni', 'p_holm', 'p_sidak', 'p_fdr'],
               dtype='object')
```

### 3.0.5 NODE4

```
In [17]: node4_df = pd.read_table('pcasom_node4_goatools_noprop.txt', skiprows=2)

         # GO Term list before limiting by significance
         print('Before significance filtering')
         print('GO Count: {}'.format(node4_df.shape[0]))
         print('Fraction of enriched genes: {:.2f}'.format(
                 node4_df[node4_df['enrichment'] == 'e'].shape[0]/node4_df.shape[0]))

         # limit GO Terms by FDR corrected p value
         node4_df = node4_df[node4_df['p_fdr'] < 0.001]

         # GO Term list before limiting by significance
         print('\nAfter significance filtering')
         print('GO Count: {}'.format(node4_df.shape[0]))
         print('Fraction of enriched genes: {:.2f}'.format(
                 node4_df[node4_df['enrichment'] == 'e'].shape[0]/node4_df.shape[0]))

         node4_df = node4_df[node4_df['enrichment'] == 'e']

         node4_df.to_csv('pcasom_node4_goenrichment.txt', sep='\t', index=False)

         df_node4_gotags = node4_df.loc[:,'id']

         node4_df.columns
```

```
Before significance filtering
GO Count: 156
Fraction of enriched genes: 0.77

After significance filtering
```

```
GO Count: 19
Fraction of enriched genes: 0.68
```

```
Out[17]: Index(['id', 'enrichment', 'description', 'ratio_in_study', 'ratio_in_pop',
               'p_uncorrected', 'p_bonferroni', 'p_holm', 'p_sidak', 'p_fdr'],
              dtype='object')
```

**Plotting the expression values of node clustered genes**

```
In [18]: ##############
         ### NODE 1 ###
         ##############
         df_node1_plot = df_node1.copy()
         print('node1 preGO: {}'.format(df_node1_plot.shape[0]))

         df_node1_plot = df_node1_plot.merge(pd.DataFrame(df_go),
                                             left_on='gene',
                                             right_index=True
                                            ).dropna()

         df_node1_plot = df_node1_plot[df_node1_plot['go'].isin(df_node1_gotags)
                                      ].loc[:,['test_id','gene']].drop_duplicates()

         print('node1 postGO: {}'.format(df_node1_plot.shape[0]))

         ##############
         ### NODE 2 ###
         ##############
         df_node2_plot = df_node2.copy()
         print('node2 preGO: {}'.format(df_node2_plot.shape[0]))

         df_node2_plot = df_node2_plot.merge(pd.DataFrame(df_go),
                                             left_on='gene',
                                             right_index=True
                                            ).dropna()

         df_node2_plot = df_node2_plot[df_node2_plot['go'].isin(df_node2_gotags)
                                      ].loc[:,['test_id','gene']].drop_duplicates()

         print('node2 postGO: {}'.format(df_node2_plot.shape[0]))

         ##############
         ### NODE 3 ###
         ##############
         df_node3_plot = df_node3.copy()
         print('node3 preGO: {}'.format(df_node3_plot.shape[0]))

         df_node3_plot = df_node3_plot.merge(pd.DataFrame(df_go),
                                             left_on='gene',
                                             right_index=True
                                            ).dropna()

         df_node3_plot = df_node3_plot[df_node3_plot['go'].isin(df_node3_gotags)
```

```
                                ].loc[:,['test_id','gene']].drop_duplicates()

        print('node3 postGO: {}'.format(df_node3_plot.shape[0]))

        ##############
        ### NODE 4 ###
        ##############
        df_node4_plot = df_node4.copy()
        print('node4 preGO: {}'.format(df_node4_plot.shape[0]))

        df_node4_plot = df_node4_plot.merge(pd.DataFrame(df_go),
                                            left_on='gene',
                                            right_index=True
                                           ).dropna()

        df_node4_plot = df_node4_plot[df_node4_plot['go'].isin(df_node4_gotags)
                                ].loc[:,['test_id','gene']].drop_duplicates()

        print('node4 postGO: {}'.format(df_node4_plot.shape[0]))
```

```
node1 preGO: 2672
node1 postGO: 474
node2 preGO: 2672
node2 postGO: 186
node3 preGO: 2914
node3 postGO: 638
node4 preGO: 3727
node4 postGO: 337
```

#### 3.0.6 Plot SOM Nodes in order

```
In [19]: df_nodeplot = pd.concat([df_node1_plot,
                                  df_node2_plot,
                                  df_node3_plot,
                                  df_node4_plot])

         df_nodeplot = df_nodeplot.merge(df_cuff_exp,
                                         left_on = 'test_id',
                                         right_index = True)
         print(df_nodeplot.shape)
```

```
(1635, 13)
```

```
In [20]: # Plot log expression values for increased contrast
         df_plot_log = df_nodeplot.drop(['test_id','gene'], axis=1).copy()
         df_plot_log = np.log2(df_plot_log + 1)

         with( sns.plotting_context( 'talk' ) ):
             plt.figure(figsize=(10,10))
             sns.set_style('darkgrid')

             g = sns.heatmap(df_plot_log, cmap='PuBuGn', yticklabels=False)
```

```
    g.set_ylabel('Gene')
    g.set_xlabel('Tissue Type')
    g.set_title('Genes Specifically Expressed in One of Eleven Tissue Types\
Organized by SOM Node')
    g.set_xticklabels(df_plot_log.columns,rotation=30)

    plt.savefig('./mesculenta_v6_output/SOM_go_enrichment_heatmap.pdf',
                bbox_inches='tight')
```

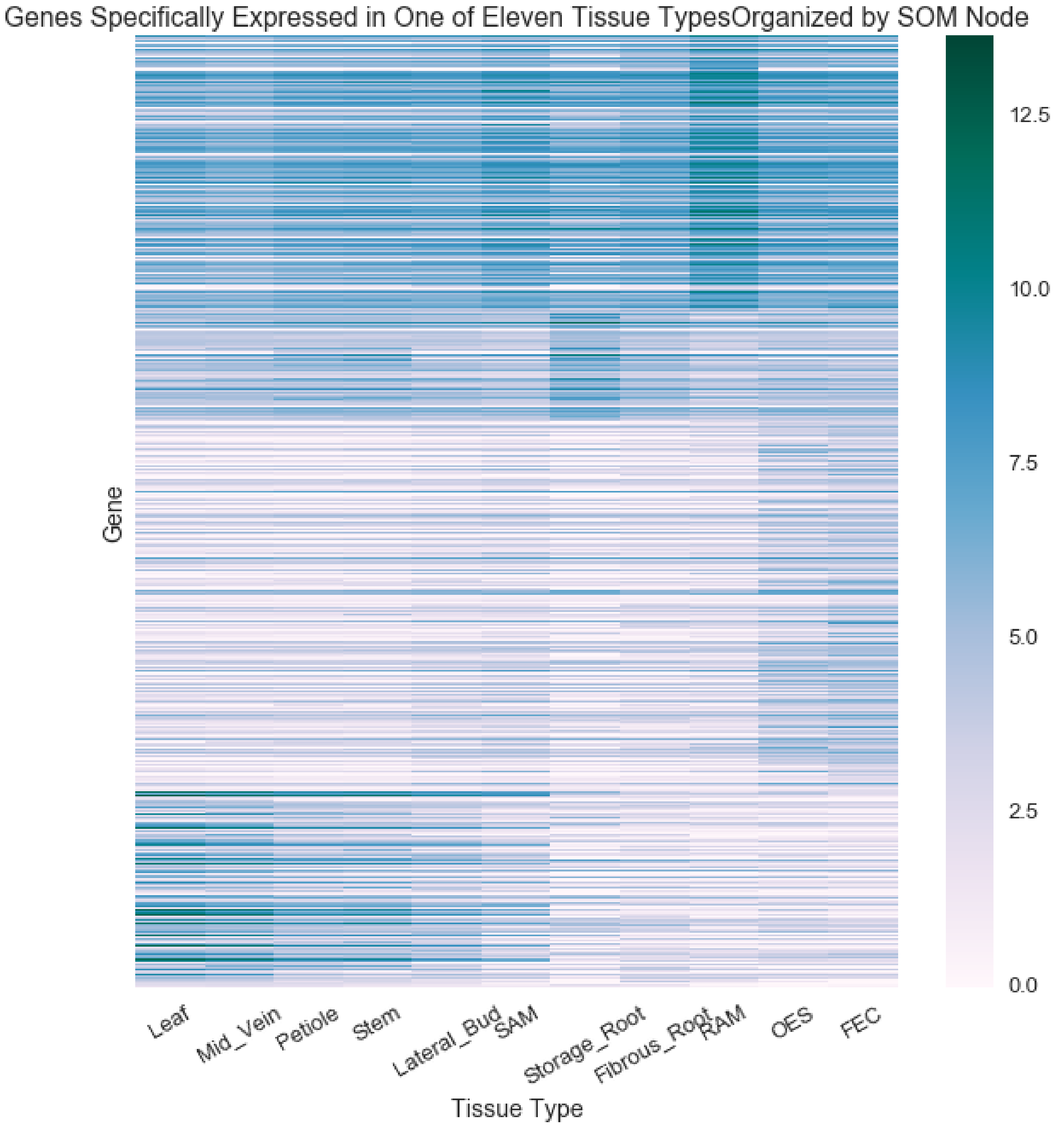

## 4 Plotting Distribution of FPKM

```
In [21]: # plot distribution of expression values in each tissue type
         with( sns.plotting_context( 'talk' ) ):
             plt.figure(figsize=(15,10))
             sns.set_style('darkgrid')

             g = sns.boxplot(data=df_cuff_ann[df_cuff_ann.iloc[:,:11] > 1].iloc[:,:11])
             g.set_yscale('log', basey=2)
             g.set_ylabel('Expression Level')
             g.set_xlabel('Tissue Type')
             g.set_title('Expression Levels of Annotated Genes \
         in Cassava in 11 Different Tissue Types (FPKM > 1)')
             g.set_xticklabels(df_cuff_ann.iloc[:,:11].columns, rotation=30)

             plt.savefig('./mesculenta_v6_output/genes_exp_dist_1FPKM.pdf',
                         bbox_inches='tight')
```

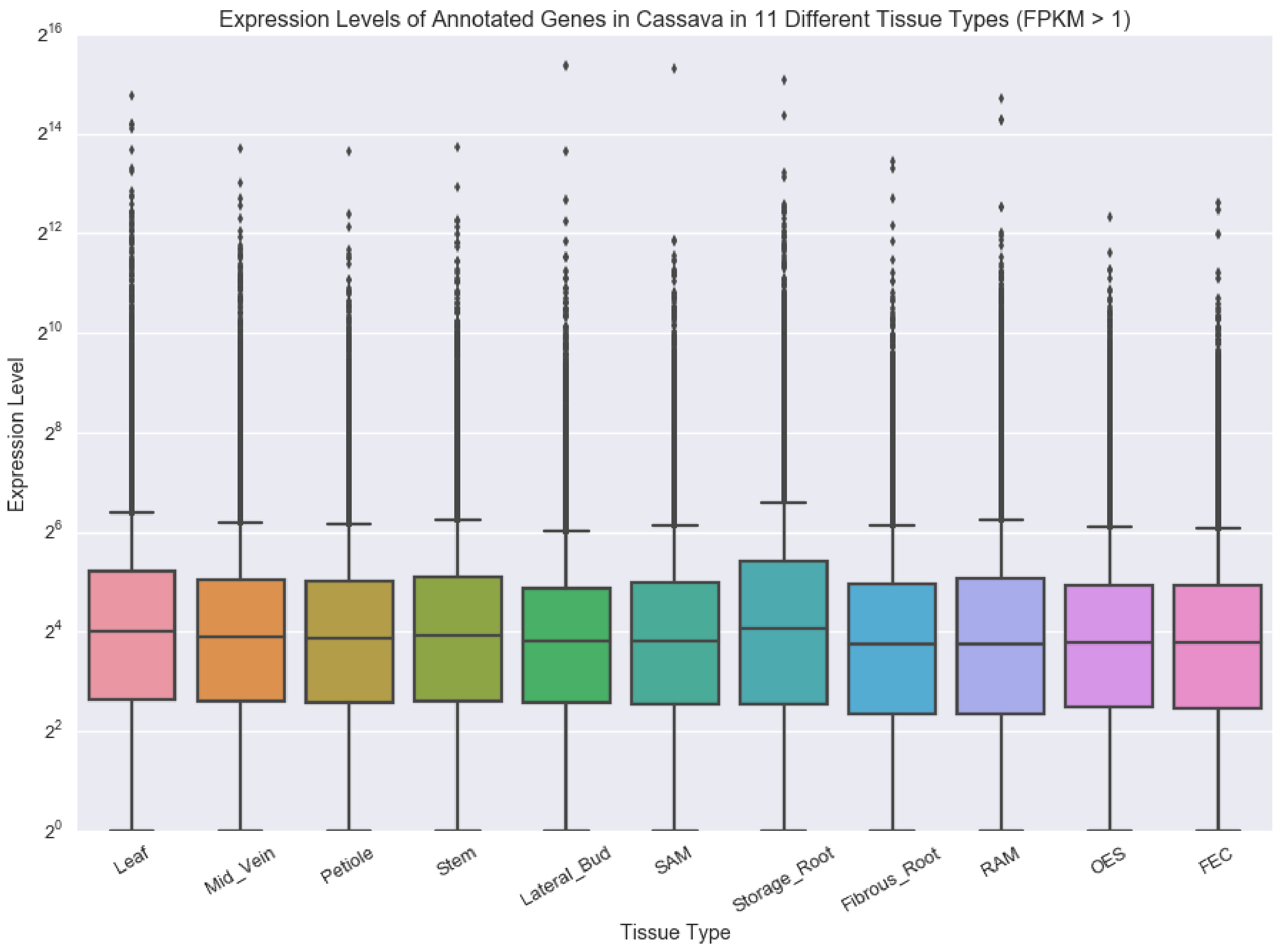


### 4.1 Gene Expression Density Plot

```
In [22]: with( sns.plotting_context( 'talk' ) ):
             sns.set_style('darkgrid')
```

```python
# plot distribution of each tissue type separately
g = sns.kdeplot(df_cuff_ann['Leaf'],clip=(0,3000), color="#33a02c",
                cumulative=True, gridsize=6000)
plt.plot((2000, df_cuff_ann['Leaf'].max()),(1,1), color="#33a02c")

sns.kdeplot(df_cuff_ann['Mid_Vein'],clip=(0,3000), color="#b2df8a",
            cumulative=True, gridsize=6000)
plt.plot((2000, df_cuff_ann['Mid_Vein'].max()),(1,1), color="#b2df8a")

sns.kdeplot(df_cuff_ann['Petiole'],clip=(0,3000), color="#1f78b4",
            cumulative=True, gridsize=6000)
plt.plot((2000, df_cuff_ann['Petiole'].max()),(1,1), color="#1f78b4")

sns.kdeplot(df_cuff_ann['Stem'],clip=(0,3000), color="#a6cee3",
            cumulative=True, gridsize=6000)
plt.plot((2000, df_cuff_ann['Stem'].max()),(1,1), color="#a6cee3")

sns.kdeplot(df_cuff_ann['Lateral_Bud'],clip=(0,3000), color="#6a3d9a",
            cumulative=True, gridsize=6000)
plt.plot((2000, df_cuff_ann['Lateral_Bud'].max()),(1,1), color="#6a3d9a")

sns.kdeplot(df_cuff_ann['SAM'],clip=(0,3000), color="#cab2d6",
            cumulative=True, gridsize=6000)
plt.plot((2000, df_cuff_ann['SAM'].max()),(1,1), color="#cab2d6")

sns.kdeplot(df_cuff_ann['Storage_Root'],clip=(0,3000), color="#ffff99",
            cumulative=True, gridsize=6000)
plt.plot((2000, df_cuff_ann['Storage_Root'].max()),(1,1), color="#ffff99")

sns.kdeplot(df_cuff_ann['Fibrous_Root'],clip=(0,3000), color="#ff7f00",
            cumulative=True, gridsize=6000)
plt.plot((2000, df_cuff_ann['Fibrous_Root'].max()),(1,1), color="#ff7f00")

sns.kdeplot(df_cuff_ann['RAM'],clip=(0,3000), color="#fdbf6f",
            cumulative=True, gridsize=6000)
plt.plot((2000, df_cuff_ann['RAM'].max()),(1,1), color="#fdbf6f")

sns.kdeplot(df_cuff_ann['OES'],clip=(0,3000), color="#e31a1c",
            cumulative=True, gridsize=6000)
plt.plot((2000, df_cuff_ann['OES'].max()),(1,1), color="#e31a1c")

sns.kdeplot(df_cuff_ann['FEC'],clip=(0,3000), color="#fb9a99",
            cumulative=True, gridsize=6000)
plt.plot((2000, df_cuff_ann['FEC'].max()),(1,1), color="#fb9a99")

g.axes.set_xlim(1,max(df_cuff_ann.max(numeric_only=True)))

# plot lines at FPKM cutoff values used in analysis
fpkm_high = 300
fpkm_on = 10
fpkm_on_loose = 8
fpkm_off = 1
```

```
    fpkm_off_loose = 4

    plt.plot((fpkm_high,fpkm_high),(0,1))
    plt.plot((fpkm_on,fpkm_on),(0,1))
    plt.plot((fpkm_on_loose,fpkm_on_loose),(0,1))
    plt.plot((fpkm_off,fpkm_off),(0,1))
    plt.plot((fpkm_off_loose,fpkm_off_loose),(0,1))

    g.set_xscale('log', basex=2)
    g.set_ylabel('Cumulative Distribution')
    g.set_xlabel('Expression Value (FPKM)')
    g.set_title('Cumulative Distribution of Annotated Gene \
Expression in 11 Tissue Types of Cassava (Log2 Scale)')

    plt.legend(loc=4)

    plt.savefig('./mesculenta_v6_output/CDF_gene_exp_logscale.pdf',
                bbox_inches='tight')
```

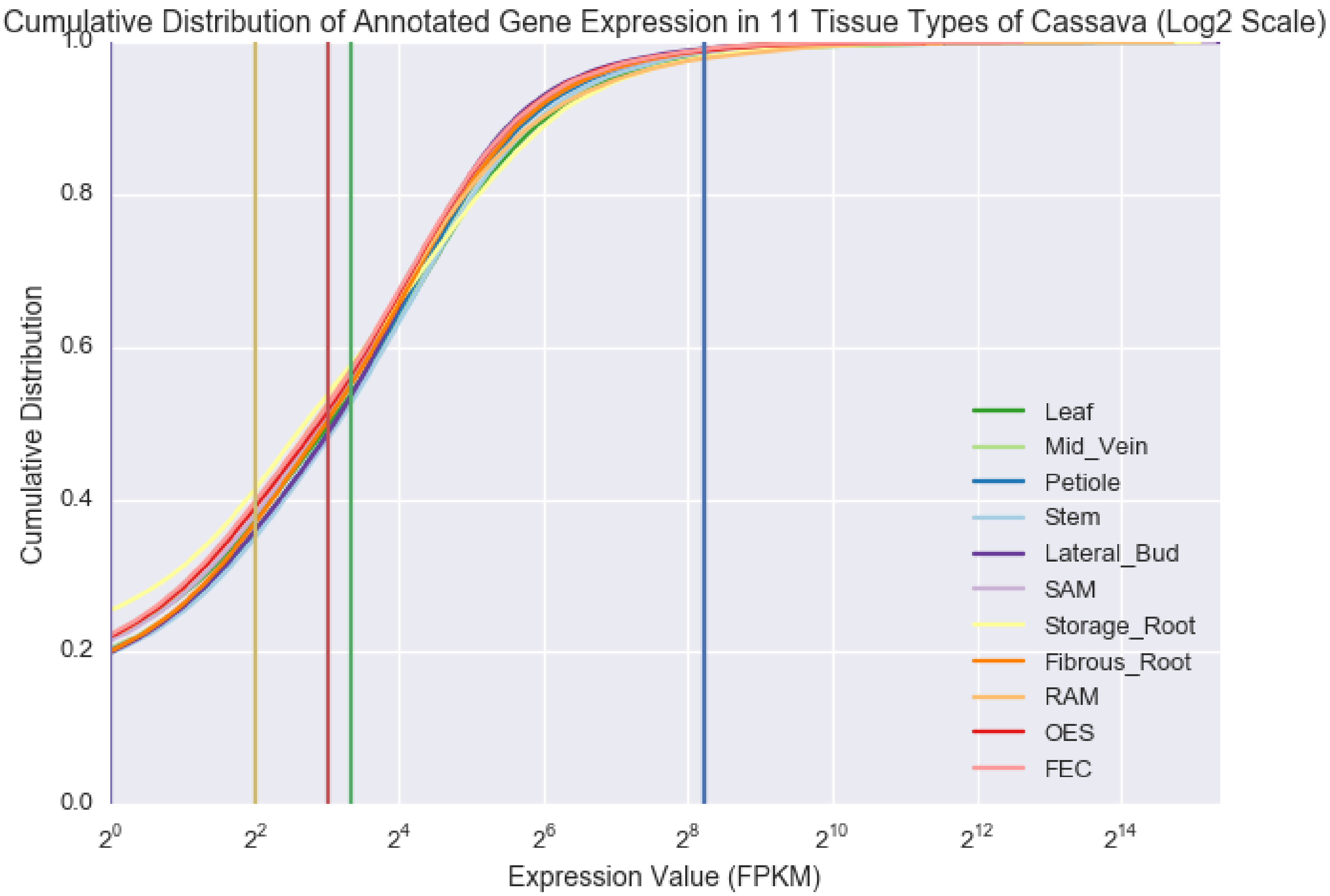


### 4.2 Percentiles of Expression Values in Tissue Types

```
In [23]: # Create dictionary of percentiles of various expression values
         perc = {i: [percentileofscore( df_cuff_ann.loc[:,[i]].values, 1, kind='weak'),
                     percentileofscore( df_cuff_ann.loc[:,[i]].values, 4, kind='weak' ),
```

```
                    percentileofscore( df_cuff_ann.loc[:,[i]].values, 8, kind='weak' ),
                    percentileofscore( df_cuff_ann.loc[:,[i]].values, 10, kind='weak' ),
                    percentileofscore( df_cuff_ann.loc[:,[i]].values, 50, kind='weak' ),
                    percentileofscore( df_cuff_ann.loc[:,[i]].values, 100, kind='weak'),
                    percentileofscore( df_cuff_ann.loc[:,[i]].values, 200, kind='weak'),
                    percentileofscore( df_cuff_ann.loc[:,[i]].values, 300, kind='weak'),
                    percentileofscore( df_cuff_ann.loc[:,[i]].values, 400, kind='weak'),
                    percentileofscore( df_cuff_ann.loc[:,[i]].values, 500, kind='weak')]
                for i in df_cuff_ann.columns[:11] }
        perc_df = pd.DataFrame(perc, index=[1,4,8,10,50,100,200,300,400,500])
        perc_df
```

Out[23]:

| | FEC | Fibrous_Root | Lateral_Bud | Leaf | Mid_Vein | OES | \ |
|---|---|---|---|---|---|---|---|
| 1 | 28.358090 | 24.136832 | 24.873561 | 28.214727 | 25.502768 | 27.947911 | |
| 4 | 41.806380 | 39.440882 | 37.879814 | 40.846641 | 38.528932 | 41.348413 | |
| 8 | 52.658198 | 50.953765 | 48.807296 | 50.049779 | 48.644021 | 51.961292 | |
| 10 | 57.078571 | 55.322369 | 53.864840 | 54.175461 | 53.076341 | 56.509100 | |
| 50 | 90.088009 | 89.128270 | 90.402612 | 86.786667 | 88.738003 | 89.992434 | |
| 100 | 95.834495 | 95.420334 | 96.133169 | 93.831389 | 95.257059 | 95.993788 | |
| 200 | 98.295568 | 98.088487 | 98.498666 | 97.284059 | 98.255744 | 98.255744 | |
| 300 | 99.092031 | 98.984509 | 99.143802 | 98.411055 | 98.936721 | 98.940703 | |
| 400 | 99.498228 | 99.386723 | 99.474334 | 98.912827 | 99.243359 | 99.342917 | |
| 500 | 99.645574 | 99.617697 | 99.657521 | 99.139819 | 99.422564 | 99.561945 | |

| | Petiole | RAM | SAM | Stem | Storage_Root |
|---|---|---|---|---|---|
| 1 | 25.032854 | 27.466051 | 27.310740 | 24.606746 | 34.184222 |
| 4 | 38.126717 | 42.069213 | 40.169647 | 37.581140 | 46.238700 |
| 8 | 48.902871 | 53.092270 | 51.308192 | 48.480745 | 54.772809 |
| 10 | 53.773247 | 57.417068 | 55.832105 | 53.048465 | 58.078133 |
| 50 | 88.690215 | 87.634901 | 89.331369 | 87.467644 | 85.767194 |
| 100 | 95.093784 | 93.731831 | 95.404404 | 94.444666 | 93.285811 |
| 200 | 97.992911 | 96.849986 | 97.881407 | 97.590697 | 96.945562 |
| 300 | 98.940703 | 97.869460 | 98.701764 | 98.729640 | 98.116363 |
| 400 | 99.334953 | 98.371232 | 99.127872 | 99.195572 | 98.649994 |
| 500 | 99.522122 | 98.677870 | 99.346900 | 99.438493 | 98.952650 |

## 4.3 Highly Expressed Genes Across All Tissue Types

**Cutoff of Same Value Determined by Housekeeping Genes (Specifically Max Expression of Manes.09G039900)**

In [24]:
```
# drop genes that have expression less than 300 in any tissue type
df_cuff_min = df_cuff_ann[df_cuff_ann.min(axis=1,numeric_only=True) > 300]

# set gene_id as index
df_cuff_min = df_cuff_min.set_index('gene_id')

print('Highly Expressed Genes: {}'.format(df_cuff_min.shape[0]))
```

```
Highly Expressed Genes: 31
```

In [25]:
```
with( sns.plotting_context( 'talk' ) ):
    plt.figure(figsize=(15,10))
    sns.set_style('darkgrid')
```

```
    g = sns.boxplot(data=df_cuff_min.iloc[:,:11])
    g.set_yscale('log', basey=2)
    g.set_ylabel('Expression Level')
    g.set_xlabel('Tissue Type')
    g.set_title('Expression Levels of Highly Expressed Annotated Genes \
in Cassava in 11 Different Tissue Types (tophat/cufflinks to v6.1)')
    g.set_xticklabels(df_cuff_min.iloc[:,:11].columns, rotation=30)

    plt.savefig('./mesculenta_v6_output/genes_high_exp_dist.pdf',
                bbox_inches='tight')
```

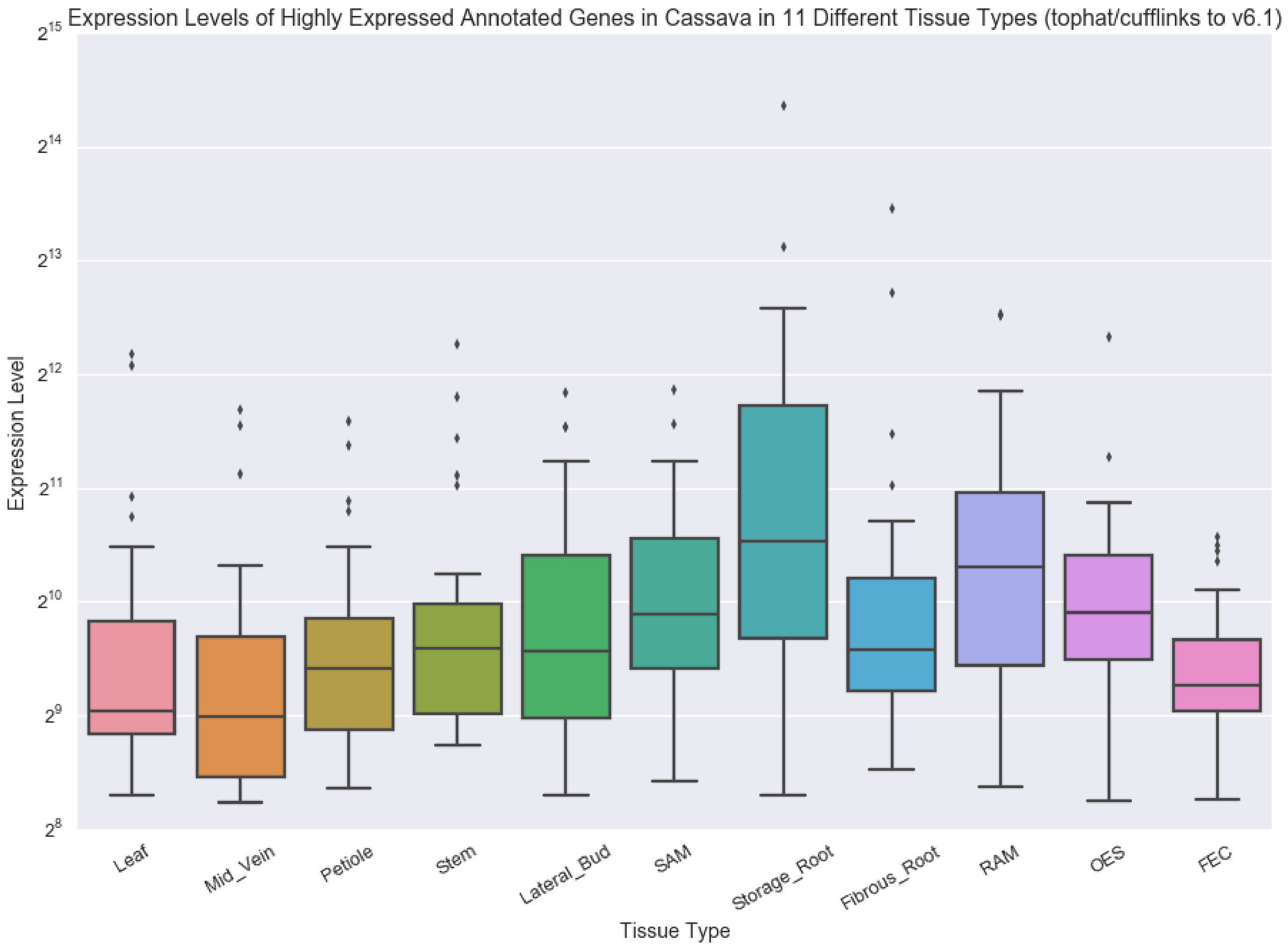


```
In [26]: df_plot = df_cuff_min.drop(['locus','annot'], axis=1).set_index(['gene'])

         with( sns.plotting_context( 'talk' ) ):
             plt.figure(figsize=(15,15))
             sns.set_style('darkgrid')

             g = sns.heatmap(df_plot,
                             cmap='Blues',
                             annot=True,
```

```
                              fmt='.0f',
                              vmax=7000   # set max for heatmap color scale at
                                          #    7000FPKM to capture more contrast
                             )

g.set_ylabel('Gene')
g.set_xlabel('Tissue Type')
plt.savefig('./mesculenta_v6_output/genes_high_exp_annot_heatmap.pdf',
            bbox_inches='tight')
```

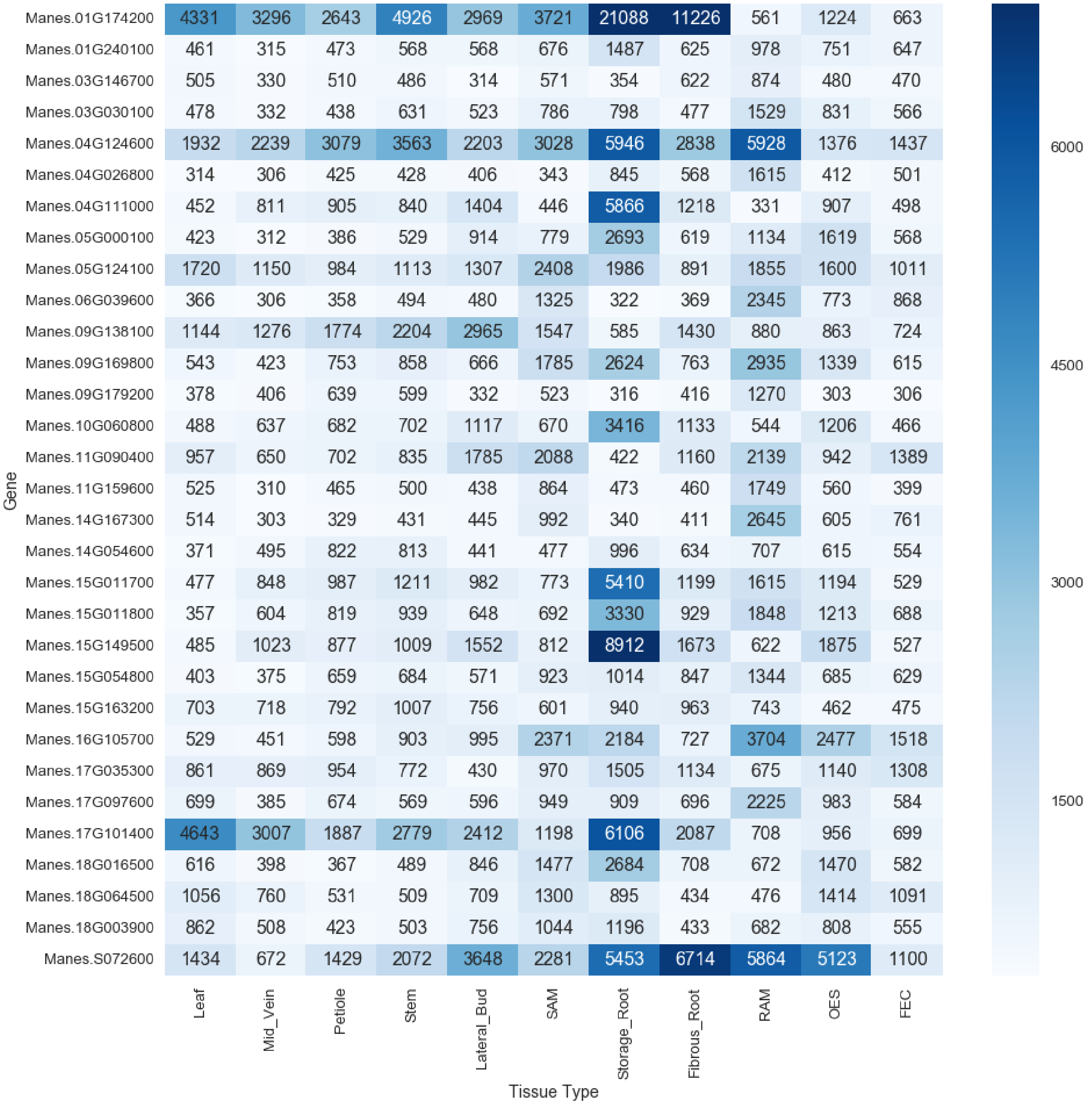


```
In [27]: # associate highly expressed genes with functional annotations
```

```python
high_exp_genes = df_cuff_min['gene']
high_exp_genes = high_exp_genes.to_frame().merge(annot.loc[:,['gene','annot']],
                                                 how='left',
                                                 on='gene'
                                                )

# sort by 'gene' and set 'gene' as index
high_exp_genes = high_exp_genes.sort_values('gene').set_index('gene')

# write to table
high_exp_genes.to_csv('./mesculenta_v6_output/high_exp_gene_annot_table.txt',
                      sep='\t')

high_exp_genes
```

Out[27]:

```
                                                      annot
gene
Manes.01G174200                            metallothionein 2B
Manes.01G240100              60S acidic ribosomal protein family
Manes.03G030100               Ribosomal protein S30 family protein
Manes.03G146700     GTP binding Elongation factor Tu family protein
Manes.04G026800                         ascorbate peroxidase 1
Manes.04G111000                               polyubiquitin 10
Manes.04G124600            Translation machinery associated TMA7
Manes.05G000100            Ribosomal protein L19e family protein
Manes.05G124100                           high mobility group B2
Manes.06G039600             Ribosomal protein L39 family protein
Manes.09G138100                          dehydrin family protein
Manes.09G169800                          Ribosomal protein S21e
Manes.09G179200                      ADP-ribosylation factor A1E
Manes.10G060800                               DNAJ homologue 2
Manes.11G090400                                 rotamase CYP 1
Manes.11G159600              60S acidic ribosomal protein family
Manes.14G054600  glyceraldehyde-3-phosphate dehydrogenase C sub...
Manes.14G167300       Zinc-binding ribosomal protein family protein
Manes.15G011700           translationally controlled tumor protein
Manes.15G011800           translationally controlled tumor protein
Manes.15G054800     GTP binding Elongation factor Tu family protein
Manes.15G149500                      ADP-ribosylation factor A1F
Manes.15G163200         cold, circadian rhythm, and rna binding 2
Manes.16G105700             Ribosomal protein S30 family protein
Manes.17G035300                                      ubiquitin 4
Manes.17G097600  translocase of the outer mitochondrial membrane 6
Manes.17G101400         cold, circadian rhythm, and RNA binding 1
Manes.18G003900                           high mobility group B2
Manes.18G016500                      Histone superfamily protein
Manes.18G064500                      Histone superfamily protein
Manes.S072600         Zinc-binding ribosomal protein family protein
```

## 4.4 Specific Tissue Expression

### Single Tissue Expression for Promoters

**tissue specific on10 off1**

```
In [28]: on_thresh = 10
         off_thresh = 1

         df_tiss_spec = pd.DataFrame( columns = df_cuff_ann.columns )

         # loop through each tissue
         for t in tissue_order:
             t = [t]

             # create index lists of on tisues and off tissues
             index_on = sorted([ i for j in tissue_order
                                if j in t for i in tissue_rep_index[j] ])
             index_off = sorted([ i for j in tissue_order
                                 if j not in t for i in tissue_rep_index[j] ])

             # subset the replicate dataset for genes matching the tissue
             #    parameters for this iteration of the loop
             df_temp = genes_rgt_piv[(genes_rgt_piv.iloc[:,index_off]
                                     < off_thresh).all(axis=1) &
                                    (genes_rgt_piv.iloc[:,index_on]
                                     > on_thresh).all(axis=1)
                                   ]

             # select for genes with annotations
             df_temp = df_temp[df_temp['gene_id'].isin(df_cuff_ann['gene_id'])]

             # add annotations and use mean expression values for each tissue
             #    instead of replicate data
             df_temp = df_temp['gene_id'].to_frame().merge(df_cuff_ann_all, on='gene_id')

             # sort by max value of each gene
             df_max = df_temp.iloc[:,:11].max(axis=1)
             df_temp = df_temp.reindex( df_max.sort_values(ascending=False).index )

             # keep only the top 3 genes in each tissue
             df_tiss_spec = pd.concat([df_tiss_spec,
                                      df_temp.iloc[:3,:]
                                     ]
                                    )

         df_tiss = df_tiss_spec.copy().set_index('gene')

         print( 'Gene Count: {}'.format(df_tiss.shape[0]) )
```

```
Gene Count: 11
```

**tissue specific on10 off1: Grouped Tissues**

```
In [29]: on_thresh = 10
         off_thresh = 1
```

```
df_tiss_spec = pd.DataFrame( columns = df_cuff_ann.columns )

tissue_groups = [['Leaf','Mid_Vein','Petiole','Stem','Lateral_Bud','SAM'],
                 ['Storage_Root'],['Fibrous_Root','RAM'],
                 ['OES','FEC']
                ]

# loop through each tissue
for t in tissue_groups:
    # create index lists of on tisues and off tissues
    index_on = sorted([ i for j in tissue_order
                       if j in t for i in tissue_rep_index[j] ])
    index_off = list( set(range(32)) - set(index_on) )

    # subset the replicate dataset for genes matching the tissue
    #    parameters for this iteration of the loop
    df_temp = genes_rgt_piv[(genes_rgt_piv.iloc[:,index_off]
                             < off_thresh).all(axis=1) &
                            (genes_rgt_piv.iloc[:,index_on]
                             > on_thresh).all(axis=1)
                           ]

    # select for genes with annotations
    df_temp = df_temp[df_temp['gene_id'].isin(df_cuff_ann['gene_id'])]

    # add annotations and use mean expression values for each tissue
    #    instead of replicate data
    df_temp = df_temp['gene_id'].to_frame().merge(df_cuff_ann, on='gene_id')

    # sort by max value of each gene
    df_max = df_temp.iloc[:,:11].max(axis=1)
    df_temp = df_temp.reindex( df_max.sort_values(ascending=False).index )

    # keep only the top 3 genes in each tissue
    df_tiss_spec = pd.concat([df_tiss_spec,
                              df_temp.iloc[:3,:]
                             ]
                            )

df_tissgrps = df_tiss_spec.copy().set_index('gene')

print( 'Gene Count: {}'.format(df_tissgrps.shape[0]) )
```

```
Gene Count: 9
```

**tissue specific on8 off4**

```
In [30]: on_thresh = 8
         off_thresh = 4

         df_tiss_spec = pd.DataFrame( columns = df_cuff_ann.columns )

         # loop through each tissue
```

```
for t in tissue_order:
    # using relaxed parameters, finding single tissue genes in
    #    each tissue without 3 genes with strict parameters
    if t == 'FEC' or t == 'Fibrous_Root' or t == 'RAM':
        continue

    t = [t]

    # create index lists of on tisues and off tissues
    index_on = sorted([ i for j in tissue_order
                       if j in t for i in tissue_rep_index[j] ])
    index_off = sorted([ i for j in tissue_order
                        if j not in t for i in tissue_rep_index[j] ])

    # subset the replicate dataset for genes matching the tissue
    #    parameters for this iteration of the loop
    df_temp = genes_rgt_piv[(genes_rgt_piv.iloc[:,index_off]
                             < off_thresh).all(axis=1) &
                            (genes_rgt_piv.iloc[:,index_on]
                             > on_thresh).all(axis=1)
                           ]

    # select for genes with annotations
    df_temp = df_temp[df_temp['gene_id'].isin(df_cuff_ann['gene_id'])]

    # add annotations and use mean expression values for each tissue
    #    instead of replicate data
    df_temp = df_temp['gene_id'].to_frame().merge(df_cuff_ann, on='gene_id')
    df_temp = df_temp.drop_duplicates('gene')

    # sort by max value of each gene
    df_max = df_temp.iloc[:,:11].max(axis=1)
    df_temp = df_temp.reindex( df_max.sort_values(ascending=False).index )

    # keep only the top 3 genes in each tissue
    df_tiss_spec = pd.concat([df_tiss_spec,
                              df_temp.iloc[:3,:]
                             ]
                            )

df_tissrlx = df_tiss_spec.copy().set_index('gene')

print( 'Gene Count: {}'.format(df_tissrlx.shape[0]) )
```

```
Gene Count: 17
```

#### Tissue Specific Plot Prep

In [31]:
```
# drop first gene in SAM so it's not duplicated in the plot
df_tissrlx.drop( df_tissrlx[df_tissrlx['SAM'] > on_thresh].index[1:],
                inplace=True)

# concatenate the 3 Tissue Specfic DataFrames for the plot
```

```python
df_plot = pd.concat( [df_tiss, df_tissrlx, df_tissgrps] )
df_plot['annot'].to_csv('./mesculenta_v6_output/specific_exp_gene_annotonly.txt')

# Restrict plot DataFrame to expression values
df_plot = df_plot.iloc[:,:11]

# Sort Columns in tissue_order as specified earlier
df_plot = df_plot.loc[:,tissue_order]
df_tiss_spec = df_tiss_spec.set_index('gene_id')

print( 'Gene Count: {}'.format(df_plot.shape[0]) )
```

```
Gene Count: 35
```

In [32]:
```python
df_plot_log = df_plot.copy()
df_plot_log = np.log2(df_plot_log + 1)

with( sns.plotting_context( 'talk' ) ):
    plt.figure(figsize=(15,15))
    sns.set_style('darkgrid')

    g = sns.heatmap(df_plot_log, cmap='PuBuGn', annot=True, fmt='.1f')

    g.set_ylabel('Gene')
    g.set_xlabel('Tissue Type')
    g.set_title('Genes Specifically Expressed in \
Eleven Tissue Types (log2 Values)')
    g.set_xticklabels(df_plot_log.columns,rotation=30)

    plt.savefig('./mesculenta_v6_output/specific_exp_mrg_strict_relaxed_log.pdf',
                bbox_inches='tight')
```

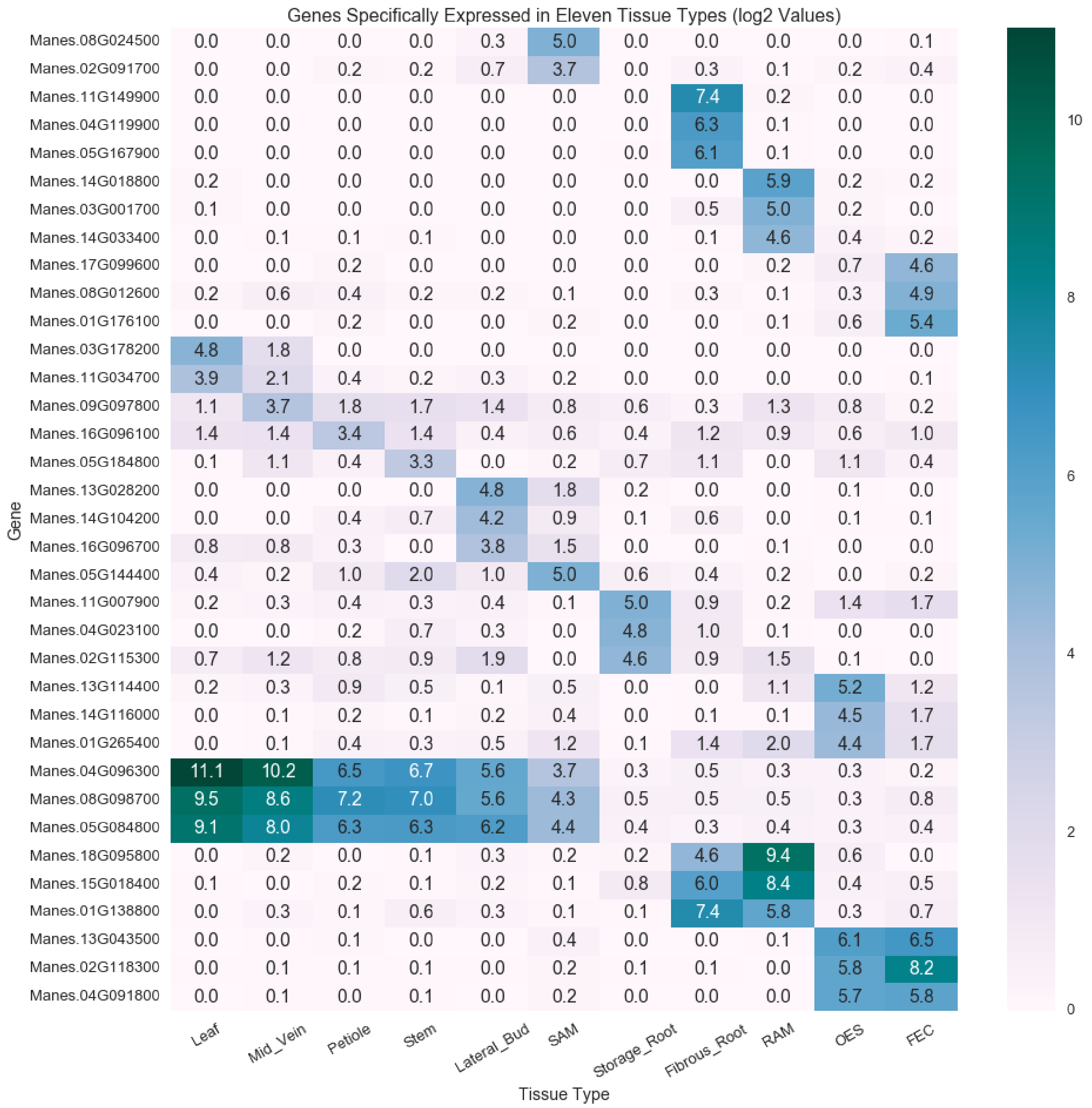


# 5 Pairwise Tissue Comparison

## 5.1 OES and FEC

### 5.1.1 Differentially Expressed in OES and FEC

```
In [33]: lfc = 2
         sig = 0.05
         fpkm_cutoff = 1
```

```
        # limit list of genes to significantly differentially
        #      expressed with a abs(log2(fold_change)) value > 2
        #      and an expression value of at least 1 FPKM in one of the tissues
        df_volc = df_cuff[(df_cuff['sample_1'] == 'FEC')
                         & (df_cuff['sample_2'] == 'OES')
                         & (df_cuff['q_value'] < sig )
                         & (np.abs(df_cuff['log2(fold_change)']) > lfc)
                         & ((df_cuff['value_1'] > fpkm_cutoff)
                            | (df_cuff['value_2'] > fpkm_cutoff))
                         & (df_cuff['gene'] != '-')].merge( annot[['gene','annot']],
                                                           how='inner',
                                                           on='gene',
                                                           copy=False)

        df_volc_n = df_cuff[(df_cuff['sample_1'] == 'FEC')
                         & (df_cuff['sample_2'] == 'OES')
                         & ((df_cuff['q_value'] >= sig )
                            | (np.abs(df_cuff['log2(fold_change)']) <= lfc ))
                         & ((df_cuff['value_1'] > fpkm_cutoff)
                            | (df_cuff['value_2'] > fpkm_cutoff))
                         & (df_cuff['gene'] != '-')].merge( annot[['gene','annot']],
                                                           how='inner',
                                                           on='gene',
                                                           copy=False)

        # write genes to file
        df_volc.to_csv('./mesculenta_v6_output/diffexp_oesfec.txt', sep='\t')

        # calculate log score for volcano plot
        df_volc['-log(q_value)'] = -np.log10(df_volc['q_value'])
        df_volc_n['-log(q_value)'] = -np.log10(df_volc_n['q_value'])

        print('OES vs FEC')
        print('Differentially Expressed Genes: {}'.format(df_volc.shape[0]))

        # Reflect foldchange values to convey the transition
        #  from OES to FEC
        df_volc['log2(fold_change)'] *=-1
        df_volc_n['log2(fold_change)'] *=-1
```

```
OES vs FEC
Differentially Expressed Genes: 2022
```

```
In [34]: ############
        ## GO PREP
        ##########
        df_volc[ df_volc['log2(fold_change)'] < -2
               ]['gene'].drop_duplicates().to_csv(
            './mesculenta_v6_output/goprep_diffexp_oesfec_oes.txt', index=False)

        df_volc[ df_volc['log2(fold_change)'] > 2
               ]['gene'].drop_duplicates().to_csv(
            './mesculenta_v6_output/goprep_diffexp_oesfec_fec.txt', index=False)
```

```
df_cuff[(df_cuff['sample_1'] == 'FEC') &
        (df_cuff['sample_2'] == 'OES') &
        ((df_cuff['value_1'] > 1) | (df_cuff['value_2'] > 1)) &
        (df_cuff['gene'] != '-')
       ]['gene'].drop_duplicates().to_csv(
    './mesculenta_v6_output/goprep_bkgrnd_oesfec.txt', index=False)
```

In [35]:
```
########################
## goatools ANALYSIS:
# python ~/src/goatools/scripts/find_enrichment.py \
#     --fdr --obo ~/src/goatools/go-basic.obo \
#     goprep_diffexp_oesfec_oes.txt goprep_bkgrnd_oesfec.txt \
#     Mesculenta_305_v6.1.annotation_info.go_only_uniq.txt \
#     > oesfec_oes_goatools.txt

## Process goatools output
### OES
print( 'OES' )
df_oesfecgo_oes = pd.read_table(
    './mesculenta_v6_output/oesfec_oes_goatools.txt', comment='#')

print( 'GO count unfiltered: {}'.format(df_oesfecgo_oes.shape[0]))
print( 'Enriched GO count, FDR < 0.01: {}'.format(
        df_oesfecgo_oes[(df_oesfecgo_oes['p_fdr'] < 0.01) &
                        (df_oesfecgo_oes['enrichment'] == 'e')
                       ].shape[0] )
     )
print( 'Enriched GO count, FDR < 0.001: {}'.format(
        df_oesfecgo_oes[(df_oesfecgo_oes['p_fdr'] < 0.001) &
                        (df_oesfecgo_oes['enrichment'] == 'e')
                       ].shape[0] )
     )

print()

########################
## goatools ANALYSIS:
# python ~/src/goatools/scripts/find_enrichment.py \
#     --fdr --obo ~/src/goatools/go-basic.obo \
#     goprep_diffexp_oesfec_fec.txt goprep_bkgrnd_oesfec.txt \
#     Mesculenta_305_v6.1.annotation_info.go_only_uniq.txt \
#     > oesfec_fec_goatools.txt

### FEC
print( 'FEC' )
df_oesfecgo_fec = pd.read_table(
    './mesculenta_v6_output/oesfec_fec_goatools.txt', comment='#')

print( 'GO count unfiltered: {}'.format(df_oesfecgo_fec.shape[0]))
print( 'Enriched GO count, FDR < 0.01: {}'.format(
        df_oesfecgo_fec[(df_oesfecgo_fec['p_fdr'] < 0.01) &
                        (df_oesfecgo_fec['enrichment'] == 'e')
```

```
                                ].shape[0] )
              )
        print( 'Enriched GO count, FDR < 0.001: {}'.format(
                df_oesfecgo_fec[(df_oesfecgo_fec['p_fdr'] < 0.001) &
                                (df_oesfecgo_fec['enrichment'] == 'e')
                               ].shape[0] )
              )
```

```
OES
GO count unfiltered: 236
Enriched GO count, FDR < 0.01: 43
Enriched GO count, FDR < 0.001: 35

FEC
GO count unfiltered: 275
Enriched GO count, FDR < 0.01: 26
Enriched GO count, FDR < 0.001: 16
```

```
In [36]: print('Genes Upregulated in FEC: {}'.format(
                 df_volc[df_volc['log2(fold_change)'] > 2].shape[0])
              )
         print('Genes Upregulated in OES: {}'.format(
                 df_volc[df_volc['log2(fold_change)'] < -2].shape[0])
              )
```

```
Genes Upregulated in FEC: 937
Genes Upregulated in OES: 1085
```

```
In [37]: lfc = 2
         on_thresh = 10
         off_thresh = 1

         with( sns.plotting_context('talk') ):
             plt.figure(figsize=(15,12))
             sns.set_style('darkgrid')


             g = sns.regplot( y='-log(q_value)',
                              x='log2(fold_change)',
                              data=df_volc_n,
                              scatter=True,
                              fit_reg=False
                            )

             g = sns.regplot( y='-log(q_value)',
                              x='log2(fold_change)',
                              data=df_volc,
                              scatter=True,
                              fit_reg=False
                            )

             y_limit = (0,4)
```

```
    x_limit = (-30,30)
    g.axes.set_ylim(*y_limit)
    g.axes.set_xlim(*x_limit)
    g.set_title( 'Volcano Plot of Genes Differentially \
Expressed from OES to FEC')

    plt.plot( (lfc,lfc), (0,y_limit[1]), color='red', alpha = 0.4 )
    plt.plot( (-lfc,-lfc), (0,y_limit[1]), color='red', alpha = 0.4 )
    plt.plot( (x_limit[0], x_limit[1]), (1.3,1.3),
              linestyle = '--', color='red', alpha = 0.4 )

    plt.savefig('./mesculenta_v6_output/volcano_diffexp_oesfec.pdf',
                bbox_inches='tight')
```

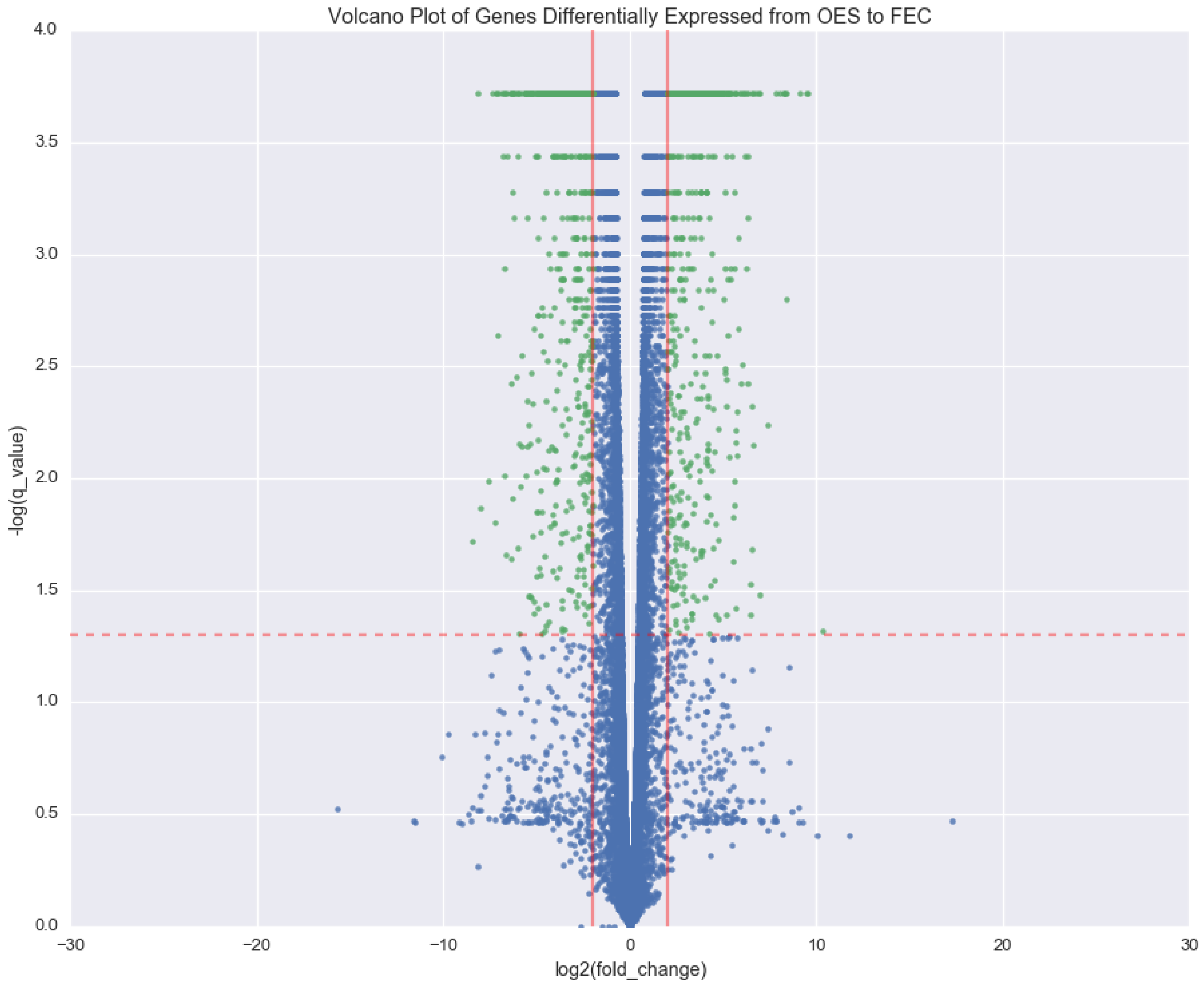

## 5.2 Storage Root and Fibrous Root

### 5.2.1 Differentially Expressed in Storage_Root and Fibrous_Root

```
In [38]: ###################
         ## ROOTS ######
         ##################

         lfc = 2
         sig = 0.05
         fpkm_cutoff = 1

         # limit list of genes to significantly differentially
         #       expressed with a abs(log2(fold_change)) value > 2
         #       and an expression value of at least 1 FPKM in one of the tissues
         df_volc = df_cuff[(df_cuff['sample_1'] == 'Storage_Root')
                           & (df_cuff['sample_2'] == 'Fibrous_Root')
                           & (df_cuff['q_value'] < sig )
                           & (np.abs(df_cuff['log2(fold_change)']) > lfc)
                           & ((df_cuff['value_1'] > fpkm_cutoff)
                              | (df_cuff['value_2'] > fpkm_cutoff))
                           & (df_cuff['gene'] != '-')].merge( annot[['gene','annot']],
                                                              how='inner',
                                                              on='gene',
                                                              copy=False)

         df_volc_n = df_cuff[(df_cuff['sample_1'] == 'Storage_Root')
                           & (df_cuff['sample_2'] == 'Fibrous_Root')
                           & ((df_cuff['q_value'] >= sig )
                              | (np.abs(df_cuff['log2(fold_change)']) <= lfc ))
                           & ((df_cuff['value_1'] > fpkm_cutoff)
                              | (df_cuff['value_2'] > fpkm_cutoff))
                           & (df_cuff['gene'] != '-')].merge( annot[['gene','annot']],
                                                              how='inner',
                                                              on='gene',
                                                              copy=False)

         # write genes to file
         df_volc.to_csv('./mesculenta_v6_output/diffexp_roots.txt', sep='\t')

         # calculate log score for volcano plot
         df_volc['-log(q_value)'] = -np.log10(df_volc['q_value'])
         df_volc_n['-log(q_value)'] = -np.log10(df_volc_n['q_value'])

         print('Storage Root vs Fibrous Root')
         print('Differentially Expressed Genes: {}'.format(df_volc.shape[0]) )
```

```
Storage Root vs Fibrous Root
Differentially Expressed Genes: 3486
```

```
In [39]: ############
         ## GO PREP
         ##########
```

```
df_volc[ df_volc['log2(fold_change)'] > 2
        ]['gene'].drop_duplicates().to_csv(
    './mesculenta_v6_output/goprep_diffexp_root_fib.txt', index=False)

df_volc[ df_volc['log2(fold_change)'] < -2
        ]['gene'].drop_duplicates().to_csv(
    './mesculenta_v6_output/goprep_diffexp_root_sto.txt', index=False)

df_cuff[(df_cuff['sample_1'] == 'Storage_Root') &
         (df_cuff['sample_2'] == 'Fibrous_Root') &
         ((df_cuff['value_1'] > 1) | (df_cuff['value_2'] > 1)) &
         (df_cuff['gene'] != '-')
        ]['gene'].drop_duplicates().to_csv(
    './mesculenta_v6_output/goprep_bkgrnd_root.txt', index=False)
```

In [40]:

```
## goatools ANALYSIS:
# python ~/src/goatools/scripts/find_enrichment.py \
#     --fdr --obo ~/src/goatools/go-basic.obo \
#     goprep_diffexp_root_fib.txt goprep_bkgrnd_root.txt \
#     Mesculenta_305_v6.1.annotation_info.go_only_uniq.txt \
#     > root_fib_goatools.txt

## Process goatools output
### FIBROUS ROOT
print('FIBROUS ROOT')
df_rootgo_fib = pd.read_table(
    './mesculenta_v6_output/root_fib_goatools.txt', comment='#')

print( 'GO count unfiltered: {}'.format(df_rootgo_fib.shape[0]))
print( 'Enriched GO count, FDR < 0.05: {}'.format(
        df_rootgo_fib[(df_rootgo_fib['p_fdr'] < 0.05) &
                      (df_rootgo_fib['enrichment'] == 'e')
                     ].shape[0] )
     )
print( 'Enriched GO count, FDR < 0.01: {}'.format(
        df_rootgo_fib[(df_rootgo_fib['p_fdr'] < 0.01) &
                      (df_rootgo_fib['enrichment'] == 'e')
                     ].shape[0] )
     )

print()

## goatools ANALYSIS:
# python ~/src/goatools/scripts/find_enrichment.py \
#     --fdr --obo ~/src/goatools/go-basic.obo \
#     goprep_diffexp_root_sto.txt goprep_bkgrnd_root.txt \
#     Mesculenta_305_v6.1.annotation_info.go_only_uniq.txt \
#     > root_sto_goatools.txt

### STORAGE ROOT
print('STORAGE ROOT')
df_rootgo_sto = pd.read_table(
    './mesculenta_v6_output/root_sto_goatools.txt', comment='#')
```

```
print( 'GO count unfiltered: {}'.format(df_rootgo_sto.shape[0]))
print( 'Enriched GO count, FDR < 0.05: {}'.format(
        df_rootgo_sto[(df_rootgo_sto['p_fdr'] < 0.05) &
                      (df_rootgo_sto['enrichment'] == 'e')
                     ].shape[0] )
     )
print( 'Enriched GO count, FDR < 0.01: {}'.format(
        df_rootgo_sto[(df_rootgo_sto['p_fdr'] < 0.01) &
                      (df_rootgo_sto['enrichment'] == 'e')
                     ].shape[0] )
     )
```

```
FIBROUS ROOT
GO count unfiltered: 414
Enriched GO count, FDR < 0.05: 139
Enriched GO count, FDR < 0.01: 135

STORAGE ROOT
GO count unfiltered: 177
Enriched GO count, FDR < 0.05: 24
Enriched GO count, FDR < 0.01: 4
```

```
In [41]: print('Genes Upregulated in Fibrous Root: {}'.format(
                 df_volc[df_volc['log2(fold_change)'] > 0].shape[0])
              )
         print('Genes Upregulated in Storage Root'.format(
                 df_volc[df_volc['log2(fold_change)'] < 0].shape[0])
              )
```

```
Genes Upregulated in Fibrous Root: 2524
Genes Upregulated in Storage Root
```

```
In [42]: ###################
         ## ROOTS ######
         ##################

         lfc = 2
         on_thresh = 10
         off_thresh = 1

         with( sns.plotting_context('talk') ):
             plt.figure(figsize=(15,12))
             sns.set_style('darkgrid')


             g = sns.regplot( y='-log(q_value)',
                              x='log2(fold_change)',
                              data=df_volc_n,
                              scatter=True,
                              fit_reg=False
                            )
```

```
    g = sns.regplot( y='-log(q_value)',
                    x='log2(fold_change)',
                    data=df_volc,
                    scatter=True,
                    fit_reg=False
                   )


    y_limit = (0,4)
    x_limit = (-30,30)
    g.axes.set_ylim(*y_limit)
    g.axes.set_xlim(*x_limit)
    g.set_title( 'Volcano Plot of Genes Differentially \
Expressed from Storage Root to Fibrous Root')

    plt.plot( (lfc,lfc), (0,y_limit[1]), color='red', alpha = 0.4 )
    plt.plot( (-lfc,-lfc), (0,y_limit[1]), color='red', alpha = 0.4 )
    plt.plot( (x_limit[0], x_limit[1]), (1.3,1.3),
             linestyle = '--', color='red', alpha = 0.4 )

    plt.savefig('./mesculenta_v6_output/volcano_diffexp_roots.pdf',
                bbox_inches='tight')
```

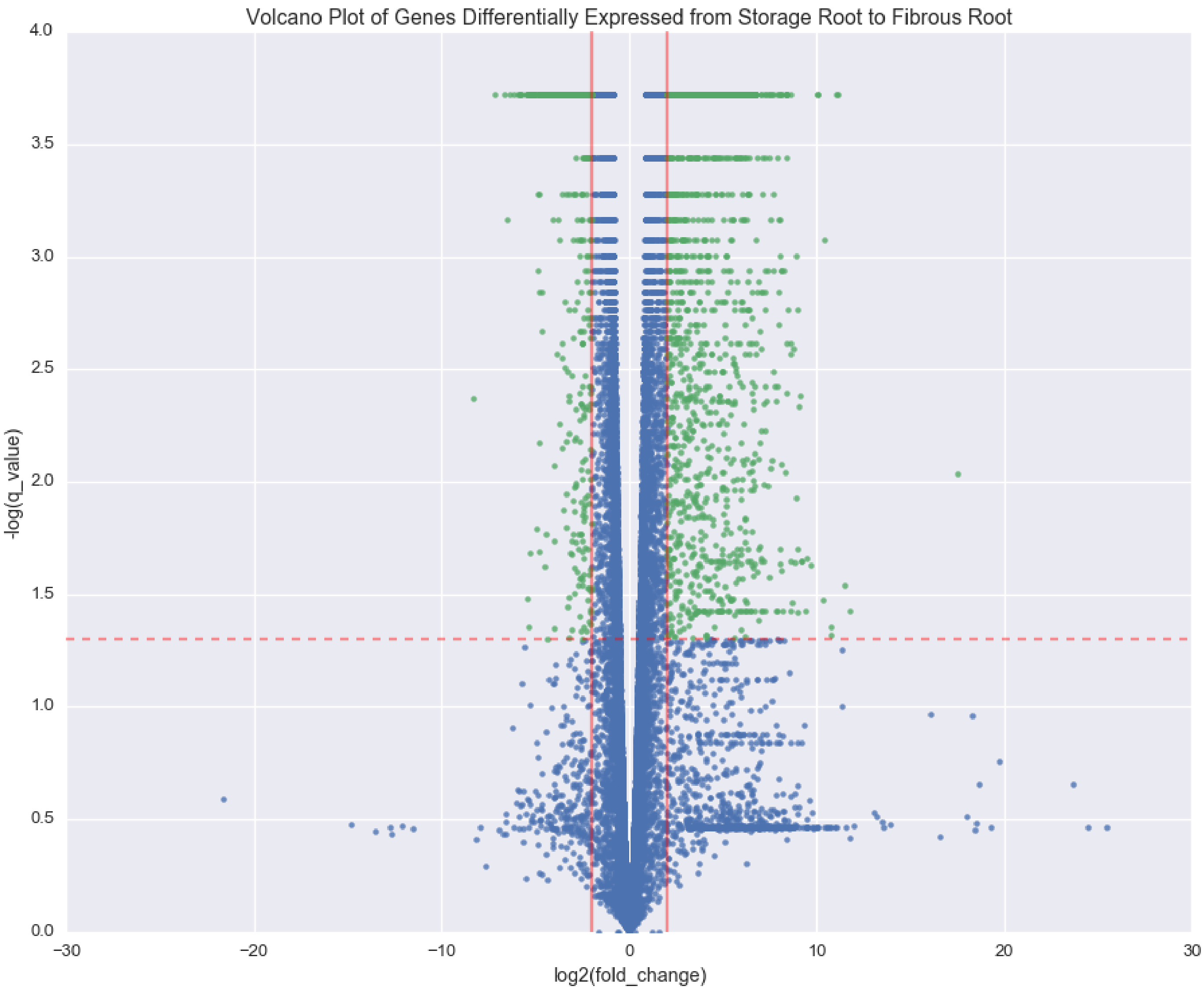


### 5.3 Fibrous Root and Leaf

#### 5.3.1 Differentially Expressed in Fibrous_Root and Leaf

In [43]:
```
##################
## ROOT/LEAF ######
##################

lfc = 2
sig = 0.05
fpkm_cutoff = 1

# limit list of genes to significantly differentially
#       expressed with a abs(log2(fold_change)) value > 2
#       and an expression value of at least 1 FPKM in one of the tissues
df_volc = df_cuff[(df_cuff['sample_1'] == 'Leaf')
                  & (df_cuff['sample_2'] == 'Fibrous_Root')
                  & (df_cuff['q_value'] < sig )
```

```
                             & (np.abs(df_cuff['log2(fold_change)']) > lfc)
                             & ((df_cuff['value_1'] > fpkm_cutoff)
                                | (df_cuff['value_2'] > fpkm_cutoff))
                             & (df_cuff['gene'] != '-')].merge( annot[['gene','annot']],
                                                                  how='inner',
                                                                  on='gene',
                                                                  copy=False)

        df_volc_n = df_cuff[(df_cuff['sample_1'] == 'Leaf')
                             & (df_cuff['sample_2'] == 'Fibrous_Root')
                             & ((df_cuff['q_value'] >= sig )
                                | (np.abs(df_cuff['log2(fold_change)']) <= lfc ))
                             & ((df_cuff['value_1'] > fpkm_cutoff)
                                | (df_cuff['value_2'] > fpkm_cutoff))
                             & (df_cuff['gene'] != '-')].merge( annot[['gene','annot']],
                                                                  how='inner',
                                                                  on='gene',
                                                                  copy=False)

        # write genes to file
        df_volc.to_csv('./mesculenta_v6_output/diffexp_rootleaf.txt', sep='\t')

        # calculate log score for volcano plot
        df_volc['-log(q_value)'] = -np.log10(df_volc['q_value'])
        df_volc_n['-log(q_value)'] = -np.log10(df_volc_n['q_value'])

        print('Leaf vs Fibrous Root')
        print('Differentially Expressed Genes: {}'.format(df_volc.shape[0]))
```

```
Leaf vs Fibrous Root
Differentially Expressed Genes: 4884
```

```
In [44]: ############
         ## GO PREP
         ##########
         df_volc[df_volc['log2(fold_change)'] > 2
                ]['gene'].drop_duplicates().to_csv(
             './mesculenta_v6_output/goprep_diffexp_rootleaf_fibroot.txt', index=False)

         df_volc[df_volc['log2(fold_change)'] < -2
                ]['gene'].drop_duplicates().to_csv(
             './mesculenta_v6_output/goprep_diffexp_rootleaf_leaf.txt', index=False)

         df_cuff[(df_cuff['sample_1'] == 'Leaf') &
                 (df_cuff['sample_2'] == 'Fibrous_Root') &
                 ((df_cuff['value_1'] > 1) | (df_cuff['value_2'] > 1)) &
                 (df_cuff['gene'] != '-')
                ]['gene'].drop_duplicates().to_csv(
             './mesculenta_v6_output/goprep_bkgrnd_rootleaf.txt', index=False)
```

```
In [45]: ##########################
         ## goatools ANALYSIS:
         # python ~/src/goatools/scripts/find_enrichment.py \
```

```
#    --fdr --obo ~/src/goatools/go-basic.obo \
#    goprep_diffexp_rootleaf_leaf.txt goprep_bkgrnd_rootleaf.txt \
#    Mesculenta_305_v6.1.annotation_info.go_only_uniq.txt \
#    > rootleaf_leaf_goatools.txt

## Process goatools output
### UPREGULATED IN LEAF
print( 'LEAF' )
df_rootgo = pd.read_table(
    './mesculenta_v6_output/rootleaf_leaf_goatools.txt', comment='#')

print( 'GO count unfiltered: {}'.format(df_rootgo.shape[0]))
print( 'Enriched GO count, FDR < 0.01: {}'.format(
        df_rootgo[(df_rootgo['p_fdr'] < 0.01) &
                  (df_rootgo['enrichment'] == 'e')
                 ].shape[0] )
     )
print( 'Enriched GO count, FDR < 0.001: {}'.format(
        df_rootgo[(df_rootgo['p_fdr'] < 0.001) &
                  (df_rootgo['enrichment'] == 'e')
                 ].shape[0] )
     )

print()

#########################
## goatools ANALYSIS:
# python ~/src/goatools/scripts/find_enrichment.py \
#    --fdr --obo ~/src/goatools/go-basic.obo \
#    goprep_diffexp_rootleaf_fibroot.txt goprep_bkgrnd_rootleaf.txt \
#    Mesculenta_305_v6.1.annotation_info.go_only_uniq.txt \
#    > rootleaf_fibroot_goatools.txt

### UPREGULATED IN FIBROUS ROOT
print( 'FIBROUS ROOT' )
df_rootgo = pd.read_table(
    './mesculenta_v6_output/rootleaf_fibroot_goatools.txt', comment='#')

print( 'GO count unfiltered: {}'.format(df_rootgo.shape[0] ))
print( 'Enriched GO count, FDR < 0.01: {}'.format(
        df_rootgo[(df_rootgo['p_fdr'] < 0.01) &
                  (df_rootgo['enrichment'] == 'e')
                 ].shape[0] )
     )
print( 'Enriched GO count, FDR < 0.001: {}'.format(
        df_rootgo[(df_rootgo['p_fdr'] < 0.001) &
                  (df_rootgo['enrichment'] == 'e')
                 ].shape[0] )
     )
```

```
LEAF
GO count unfiltered: 398
Enriched GO count, FDR < 0.01: 67
```

```
Enriched GO count, FDR < 0.001: 47

FIBROUS ROOT
GO count unfiltered: 408
Enriched GO count, FDR < 0.01: 97
Enriched GO count, FDR < 0.001: 88
```

```
In [46]: print('Genes Upregulated in Fibrous Root: {}'.format(
                 df_volc[df_volc['log2(fold_change)'] > 0].shape[0])
              )
         print('Genes Upregulated in Leaf: {}'.format(
                 df_volc[df_volc['log2(fold_change)'] < 0].shape[0])
              )
```

```
Genes Upregulated in Fibrous Root: 2446
Genes Upregulated in Leaf: 2438
```

```
In [47]: ###################
         ## ROOT/LEAF ######
         ##################

         lfc = 2
         on_thresh = 10
         off_thresh = 1

         with( sns.plotting_context('talk') ):
             plt.figure(figsize=(15,12))
             sns.set_style('darkgrid')


             g = sns.regplot( y='-log(q_value)',
                             x='log2(fold_change)',
                             data=df_volc_n,
                             scatter=True,
                             fit_reg=False
                            )

             g = sns.regplot( y='-log(q_value)',
                             x='log2(fold_change)',
                             data=df_volc,
                             scatter=True,
                             fit_reg=False
                            )

             y_limit = (0,4)
             x_limit = (-30,30)
             g.axes.set_ylim(*y_limit)
             g.axes.set_xlim(*x_limit)
             g.set_title( 'Volcano Plot of Genes Differentially \
         Expressed from Leaf to Fibrous Root')

             plt.plot( (lfc,lfc), (0,y_limit[1]), color='red', alpha = 0.4 )
```

```
    plt.plot( (-lfc,-lfc), (0,y_limit[1]), color='red', alpha = 0.4 )
    plt.plot( (x_limit[0], x_limit[1]), (1.3,1.3),
              linestyle = '--', color='red', alpha = 0.4 )

    plt.savefig('./mesculenta_v6_output/volcano_diffexp_rootleaf.pdf',
                bbox_inches='tight')
```

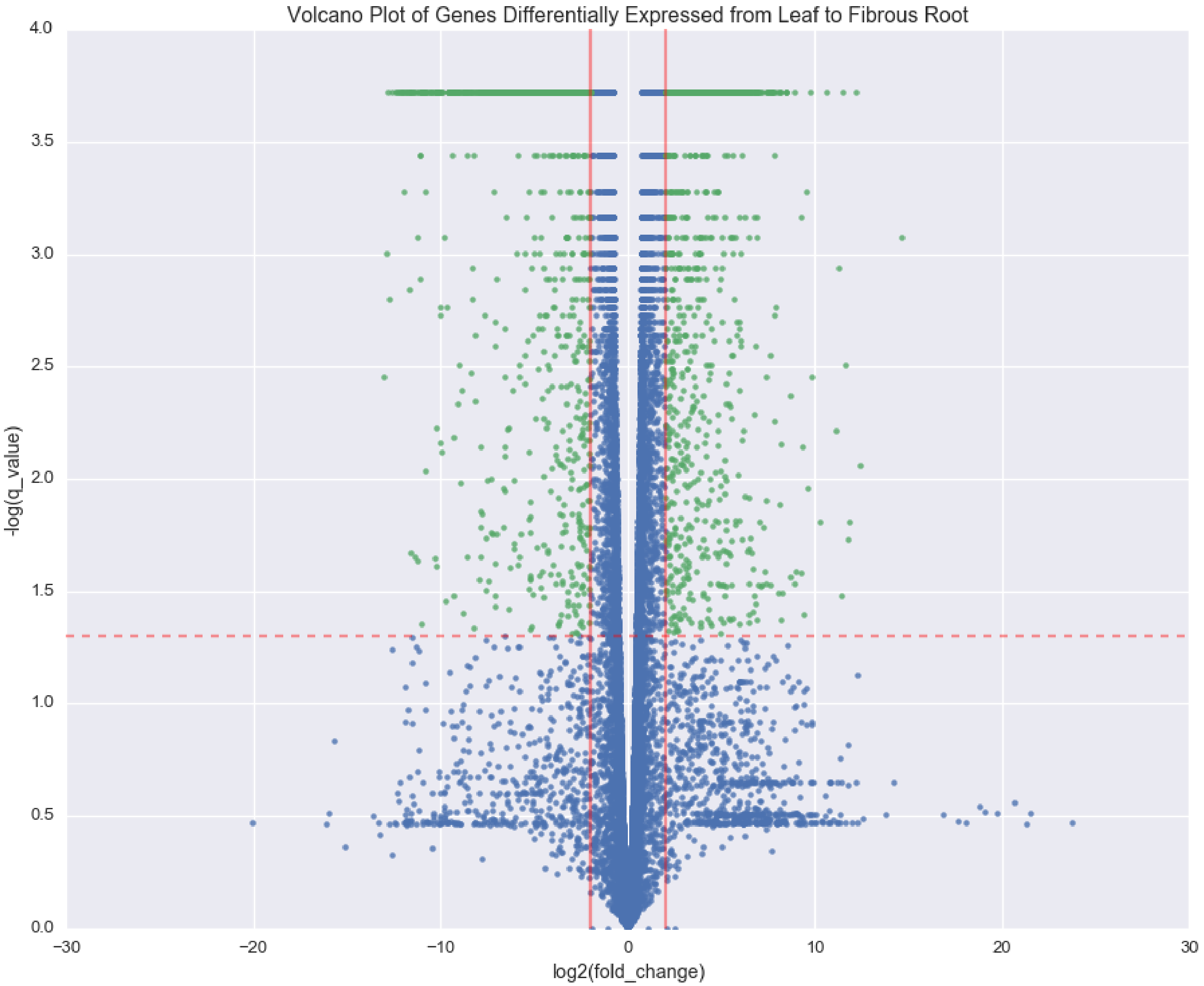


## 6 Similarly Expressed Genes

```
In [48]: genes_rgt_sim = genes_rgt_piv.copy()

         # limit to genes with a minimum expression of 40 FPKM in all samples
         genes_rgt_sim = genes_rgt_sim[(genes_rgt_sim.min(axis=1) >= 40)]

         # calculate Coefficient of Variation
         genes_rgt_sim['CoV'] = genes_rgt_sim.std(axis=1) / genes_rgt_sim.mean(axis=1)
```

```
print( 'Gene Count: {}'.format(genes_rgt_sim.shape[0]) )

# display top ten genes sorted by Coefficient of Variation
genes_rgt_sim.sort_values('CoV').columns
```

```
Gene Count: 994
```

```
Out[48]: Index(['FEC0', 'FEC1', 'FEC2', 'Fibrous_Root0', 'Fibrous_Root1',
                'Fibrous_Root2', 'Lateral_Bud0', 'Lateral_Bud1', 'Lateral_Bud2',
                'Leaf0', 'Leaf1', 'Leaf2', 'Mid_Vein0', 'Mid_Vein1', 'Mid_Vein2',
                'OES0', 'OES1', 'OES2', 'Petiole0', 'Petiole1', 'Petiole2', 'RAM0',
                'RAM1', 'RAM2', 'SAM0', 'SAM1', 'SAM2', 'Stem0', 'Stem1', 'Stem2',
                'Storage_Root0', 'Storage_Root1', 'gene', 'go', 'TAIR', 'annot',
                'gene_id', 'CoV'],
               dtype='object', name='pivot')
```

```
In [49]: # get functional annotations for top ten similarly
         #    expressed as sorted by Coefficient of Variation
         sim_genes = genes_rgt_sim.sort_values('CoV').head(10)['gene']
         sim_genes = sim_genes.to_frame().merge(annot.loc[:,['gene','annot']],
                                                 how='left', on='gene')

         sim_genes
```

Out[49]:

| | gene | annot |
|---|---|---|
| 0 | Manes.01G240900 | RNA-binding (RRM/RBD/RNP motifs) family protein |
| 1 | Manes.01G054500 | subunit of exocyst complex 8 |
| 2 | Manes.06G055400 | SIT4 phosphatase-associated family protein |
| 3 | Manes.02G019200 | PLAC8 family protein |
| 4 | Manes.06G073900 | decapping 5 |
| 5 | Manes.16G049900 | cytochrome c oxidase assembly protein CtaG / C... |
| 6 | Manes.16G093200 | RNA-binding (RRM/RBD/RNP motifs) family protein |
| 7 | Manes.11G162700 | Transducin/WD40 repeat-like superfamily protein |
| 8 | Manes.09G156800 | Sec23/Sec24 protein transport family protein |
| 9 | Manes.10G094300 | NaN |

```
In [50]: # select expression values for 3 previously used housekeeping genes
         genes_rgt_hskp = pd.concat([
                 genes_rgt_sim.sort_values('CoV').head(10).drop('CoV', axis=1),
                 genes_rgt_piv[genes_rgt_piv['gene'].isin(['Manes.07G019300',
                                                           'Manes.09G086600',
                                                           'Manes.09G039900']
                                                         )]
                                   ])

         # calculate Coefficient of Variation
         genes_rgt_hskp['CoV'] = genes_rgt_hskp.std(axis=1) / genes_rgt_hskp.mean(axis=1)
         genes_rgt_hskp = genes_rgt_hskp.sort_values('CoV')

         # write to file
         genes_rgt_hskp.to_csv(
             './mesculenta_v6_output/similar_exp_gene_annot_table.txt', sep='\t')
```

```
        genes_rgt_hskp.loc[:,['annot']].to_csv(
            './mesculenta_v6_output/similar_exp_gene_annotonly.txt', sep='\t')

        genes_rgt_hskp.columns

Out[50]: Index(['FEC0', 'FEC1', 'FEC2', 'Fibrous_Root0', 'Fibrous_Root1',
                'Fibrous_Root2', 'Lateral_Bud0', 'Lateral_Bud1', 'Lateral_Bud2',
                'Leaf0', 'Leaf1', 'Leaf2', 'Mid_Vein0', 'Mid_Vein1', 'Mid_Vein2',
                'OES0', 'OES1', 'OES2', 'Petiole0', 'Petiole1', 'Petiole2', 'RAM0',
                'RAM1', 'RAM2', 'SAM0', 'SAM1', 'SAM2', 'Stem0', 'Stem1', 'Stem2',
                'Storage_Root0', 'Storage_Root1', 'gene', 'go', 'TAIR', 'annot',
                'gene_id', 'CoV'],
               dtype='object', name='pivot')

In [51]: # get top 10 genes sorted by Coefficient of Variation for plotting
         df_plot = genes_rgt_hskp.set_index('gene').iloc[:,:32]

         with( sns.plotting_context( 'talk' ) ):
             plt.figure(figsize=(25,6))
             sns.set_style('darkgrid')

             g = sns.heatmap(data=df_plot,
                             cmap='Blues', annot=True, fmt='.0f', vmax=300)

             g.set_ylabel('Gene')
             g.set_xlabel('Tissue Type')
             g.set_title('Expression of Proposed and Used Housekeeping \
         Genes in Various Tissue Types')
```

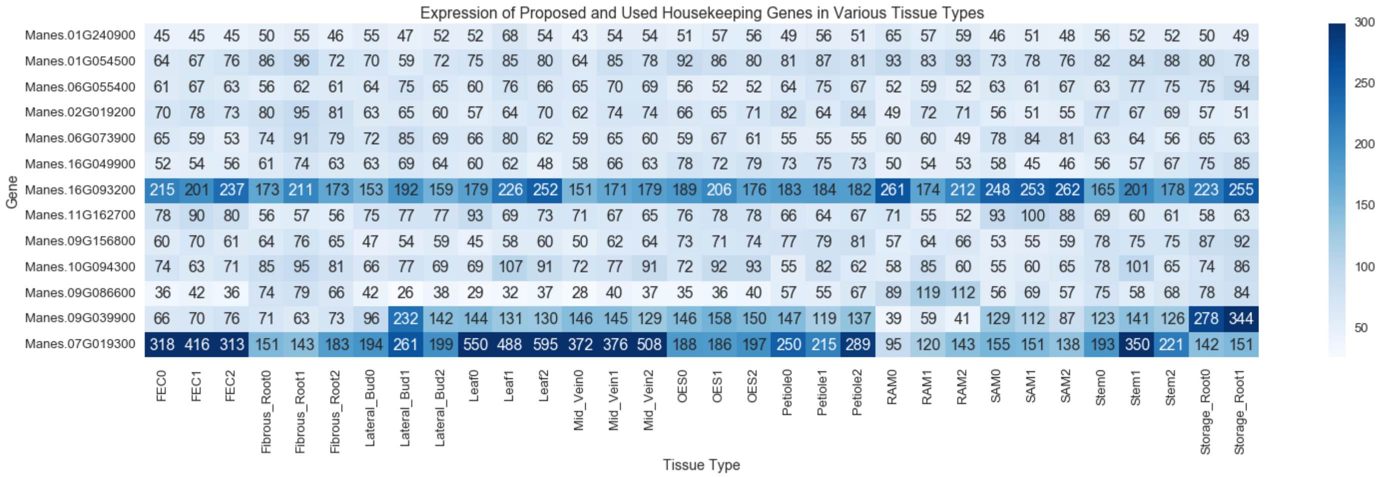


```
In [52]: df_plot = genes_rgt_hskp.copy()
         df_plot = df_plot.drop(['annot',
                                 'TAIR',
                                 'go',
                                 'gene_id',
                                 'CoV'], axis=1).set_index('gene')

         # plot distribution of gene expression across all samples of top most
```

```
#     similarly expressed genes and 3 previously used housekeeping genes
with( sns.plotting_context( 'talk' ) ):
    plt.figure(figsize=(15,10))
    sns.set_style('darkgrid')

    g = sns.boxplot(data=df_plot.transpose())
    g.set_ylabel('Expression')
    g.set_xlabel('Gene')
    g.set_title('Distribution of Expression Values of Potential \
Housekeeping Genes Across 11 Tissue Types')
    g.set_xticklabels(df_plot.index, rotation=40)

    plt.savefig('./mesculenta_v6_output/similar_exp_allsamp_cov_dist.pdf',
                bbox_inches='tight')
```

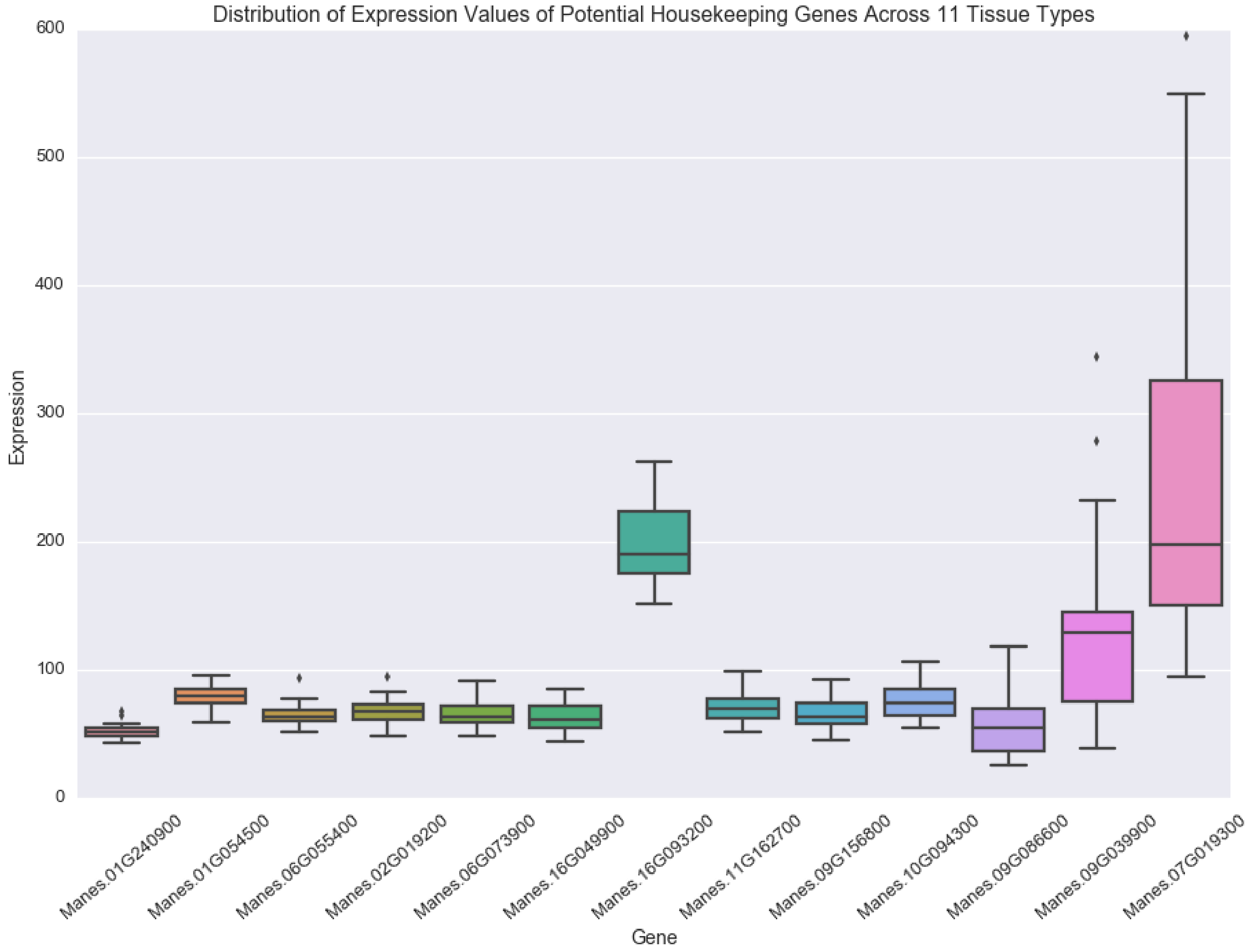


```
In [ ]:
```
